# TLR9‐Driven S‐Palmitoylation in Dendritic Cells Reveals Immune and Metabolic Protein Targets

**DOI:** 10.1002/eji.70039

**Published:** 2025-08-19

**Authors:** Juan N. Quiroz, Malte Sielaff, Daria Kondrateva, Fatima Boukhallouk, Gloria J. Godoy, Cecilia R. Molina, Brecht Moonen, Claudia C. Motran, Jeroen Bogie, Hugo D. Luján, Stefan Tenzer, Tim Sparwasser, Luciana Berod

**Affiliations:** ^1^ Institute of Molecular Medicine University Medical Center of the Johannes Gutenberg‐University Mainz Mainz Germany; ^2^ Departamento De Bioquímica Clínica Facultad De Ciencias Químicas Universidad Nacional de Córdoba Córdoba Argentina; ^3^ Centro De Investigaciones en Bioquímica Clínica e Inmunología (CIBICI) Consejo Nacional de Investigaciones Científicas y Técnicas (CONICET) Córdoba Argentina; ^4^ Institute of Immunology University Medical Center of the Johannes Gutenberg‐University Mainz Mainz Germany; ^5^ Research Center for Immunotherapy (FZI) University Medical Center Mainz Mainz Germany; ^6^ Institute of Medical Microbiology and Hygiene University Medical Center of the Johannes Gutenberg‐University Mainz Mainz Germany; ^7^ Hasselt University, Department of Immunology and Infection, Biomedical Research Institute Diepenbeek Belgium; ^8^ Helmholtz Institute for Translational Oncology Mainz (HI‐TRON Mainz) ‐ A Helmholtz Institute of the DKFZ Mainz Germany; ^9^ German Cancer Research Center (DKFZ) Heidelberg Germany

**Keywords:** dendritic cells, innate immunity, S‐palmitoylation, TLR9 signaling

## Abstract

Dendritic cells (DCs) rely on Toll‐like receptor 9 (TLR9) to detect unmethylated CpG motifs in microbial DNA, triggering essential immune responses. While the downstream signaling pathways of TLR9 activation are well characterized, their impact on S‐palmitoylation is unknown. S‐palmitoylation, involving the reversible attachment of palmitic acid to cysteine residues, plays a crucial role in regulating protein function and is catalyzed by the ZDHHC family of palmitoyl‐acyltransferases (PATs). In this study, we investigated the S‐palmitoylated proteome of bone marrow‐derived GM‐CSF DCs (GM‐DCs) at resting and following TLR9 activation with CpGB. Using the click‐chemistry‐compatible analog 17‐octadecynoic acid (17‐ODYA) and mass spectrometry (MS)‐based proteomics, we characterized dynamic remodeling of S‐palmitoylation in response to TLR9 activation. This included enrichment of targets involved in immune and metabolic pathways. Transcriptomic analysis of mice and human DCs revealed TLR9‐driven modulation of PAT‐encoding genes. Subsequently, we explored the contribution of *Zdhhc9* expression to the regulation of S‐palmitoylation in DCs. Using gene knockout approaches, we identified candidate protein targets potentially linked to ZDHHC9 activity. Interestingly, modulation of *Zdhhc9* expression alone did not influence DC maturation, suggesting that other PATs might compensate for its activity. Together, our findings reveal a novel layer of regulation in TLR9 signaling mediated by S‐palmitoylation.

## Graphical abstract

Click chemistry–based proteomics revealed dynamic remodeling of the S‐palmitoylated proteome in dendritic cells upon TLR9 activation. Transcriptomics identified TLR9‐driven regulation of ZDHHC genes, including *Zdhhc9*. Genetic approaches uncovered potential ZDHHC9‐dependent targets. Functional studies exposed key methodological limitations in investigating S‐palmitoylation in dendritic cells.

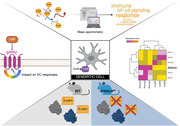

**Abbreviations**
17‐ODYA: 17‐octadecynoic acid
2‐BP: 2‐bromopalmitate
AMBIC: ammonium bicarbonate
BM: bone marrow
BMM: bone marrow macrophage
cDC: conventional dendritic cell
CTV: cell trace violet
DC: dendritic cell
DTT: dithiothreitol
ELISA: enzyme‐linked immunosorbent assay
FA: fatty acid
FACS: flow cytometry
FDR: false discovery rate
Flt3L‐DC: bone marrow‐derived Flt3L dendritic cell
GM‐DC: bone marrow‐derived GM‐CSF dendritic cell
gMFI: geometric mean fluorescence intensity
IL‐12: interleukin‐12
IL‐1α: interleukin‐1 alpha
IL‐1β: interleukin‐1 beta
IL‐6: interleukin‐6
IPA: ingenuity pathway analysis
IRF: interferon regulatory factors
LFQ: label‐free quantification
Log_2_FC: log_2_ fold change
MAPK: mitogen‐activated protein kinase
MS: mass spectrometry
NF‐κB: nuclear factor‐κB
OD: optical density
ON: overnight
PAT: palmitoyl‐acyltransferase
pDC: plasmacytoid dendritic cell
pLN: peripheral lymph node
PTM: posttranslational modification
qPCR: real‐time PCR
RT: room temperature
SD: standard deviation
SPLN: spleen
Thy: thymus
TLR: Toll‐like receptor
TNFα: tumor necrosis factor‐alpha
WT: wild‐type

## 1 Introduction

Dendritic cells (DCs) recognize unmethylated CpG DNA motifs through Toll‐like receptor 9 (TLR9) [1], a key sensor in the immune defense against viruses and bacteria [2]. Given the importance of TLR9 in mediating DC functions, several regulatory mechanisms exist to finely tune its activation, including receptor expression, compartmentalization, and signal transduction [3]. Although TLR9 expression is higher in plasmacytoid DCs (pDCs) compared with conventional DCs (cDCs), both subsets can respond to TLR9 ligands [4, 5]. In this context, we demonstrated that TLR9–MyD88 signaling is essential for cDC‐mediated control of cytomegalovirus infection [6]. Beyond protein expression, the appropriate delivery of TLR9 from the endoplasmic reticulum to endosomes is crucial for ligand recognition and downstream signaling [7]. Upon ligand binding, TLR9 mediates the activation of at least three major signaling pathways that mediate DC maturation: the mitogen‐activated protein kinase (MAPK), the nuclear factor‐κB (NF‐κB), and the interferon regulatory factors (IRFs) signaling pathways [8]. MAPK and NF‐κB converge to induce proinflammatory cytokine production, including interleukin‐6 (IL‐6), interleukin‐12 (IL‐12), and tumor necrosis factor‐alpha (TNF‐α), while simultaneously enhancing the surface expression of co‐stimulatory molecules such as CD80 and CD86 [10]. Additionally, MyD88‐dependent activation of IRF7 is crucial for inducing type I interferon secretion in response to TLR9 agonists [10]. Together, these events play a critical role in antigen presentation via the major histocompatibility complex (MHC‐I and MHC‐II), the polarization of TH1 responses, and antiviral defense [9].

Given that nearly 40% of the human proteome undergoes lipidation, this posttranslational modification (PTM) has emerged as an additional layer in the regulatory mechanisms that orchestrate immune responses [11]. Lipidation refers to the covalent attachment of fatty acids (FAs; typically, 12–20 carbon atoms in length), isoprenoids, or cholesterol groups to specific amino acid residues within proteins. The most frequent form of lipidation involves the reversible attachment of palmitic acid (C16:0) to cysteine residues via thioester linkage, known as S‐palmitoylation [12]. The addition of palmitic acid is mediated by palmitoyl‐acyltransferases (PATs), belonging to the zinc finger *Asp‐His‐His‐Cys* motif‐containing (ZDHHC) family [13], which comprises 23 proteins in mammals. Although our understanding of S‐palmitoylation regulation is still evolving, evidence suggests that both ZDHHC activity and the availability of FAs may influence this process [12]. Notably, we observed significant changes in intracellular palmitic acid pools following DC stimulation with either mycobacteria or the TLR9 agonist CpGB [14]. However, the final contribution of TLR9 activation to the regulation of S‐palmitoylation in DCs remains unexplored.

S‐palmitoylation influences protein trafficking and interactions [15], impacting several immune‐related targets. This includes components of the TLR signaling pathway, such as TLR9, TLR2, and MyD88, important surface co‐receptors like CD4 and CD86, and key immune effector proteins such as IFITM3 [16, 17]. Methodological advances in the field of quantitative proteomics have facilitated the discovery of new S‐palmitoylated proteins [18]. In this context, the use of clickable palmitic acid analogs, such as 17‐octadecynoic acid (17‐ODYA), has enabled the dynamic isolation of S‐palmitoylated proteins and subsequent identification and quantification through mass spectrometry (MS)‐based proteomics [18]. This is complemented by studies characterizing ZDHHC substrate specificity through modulation of PAT activity or expression [19]. In this context, the S‐palmitoylation of HRAS and NRAS by ZDHHC9 was among the first to be described [20]. However, given that DCs represent only 0.5%–1% of the total cell population in tissues, the direct application of these techniques in native tissues remains challenging. To overcome this limitation, different in vitro culture strategies have been established to study DC responses [21], including the generation of bone marrow‐derived DCs with GM‐CSF (GM‐DCs) and the use of TLR ligands to induce cell maturation.

In this study, we investigated the repertoire of S‐palmitoylated proteins in GM‐DCs both at rest and following TLR9 activation with CpGB. Additionally, we analyzed the expression of ZDHHC‐coding genes in DCs from both mice and humans. Finally, we explored the specific role of ZDHHC9 in modulating the S‐palmitoylated proteome and DC functional responses. Overall, this work uncovered TLR9‐mediated remodeling of S‐palmitoylated proteins involved in immune responses and cellular metabolism, coinciding with differential regulation of PAT gene expression. Notably, the S‐palmitoylated profile of some of those targets was affected by *Zdhhc9* genetic depletion, unmasking potential ZDHHC9‐restricted substrates. Finally, we demonstrated that modulating the expression of *Zdhhc9* alone does not impact DC maturation.

## 2 Results

### 2.1 GM‐DCs Display Homeostatic S‐Palmitoylation of Immune‐Related Proteins

As antigen‐presenting cells, DCs are equipped with a wide array of proteins necessary to link innate and adaptive immune responses [22]. This includes TLRs, antigen‐presenting and co‐stimulatory molecules, as well as cytokines. In this context, the characterization of homeostatic S‐palmitoylation remodeling in DCs may provide insights into additional regulatory mechanisms that control the proper activation of immune responses.

**FIGURE 1:**
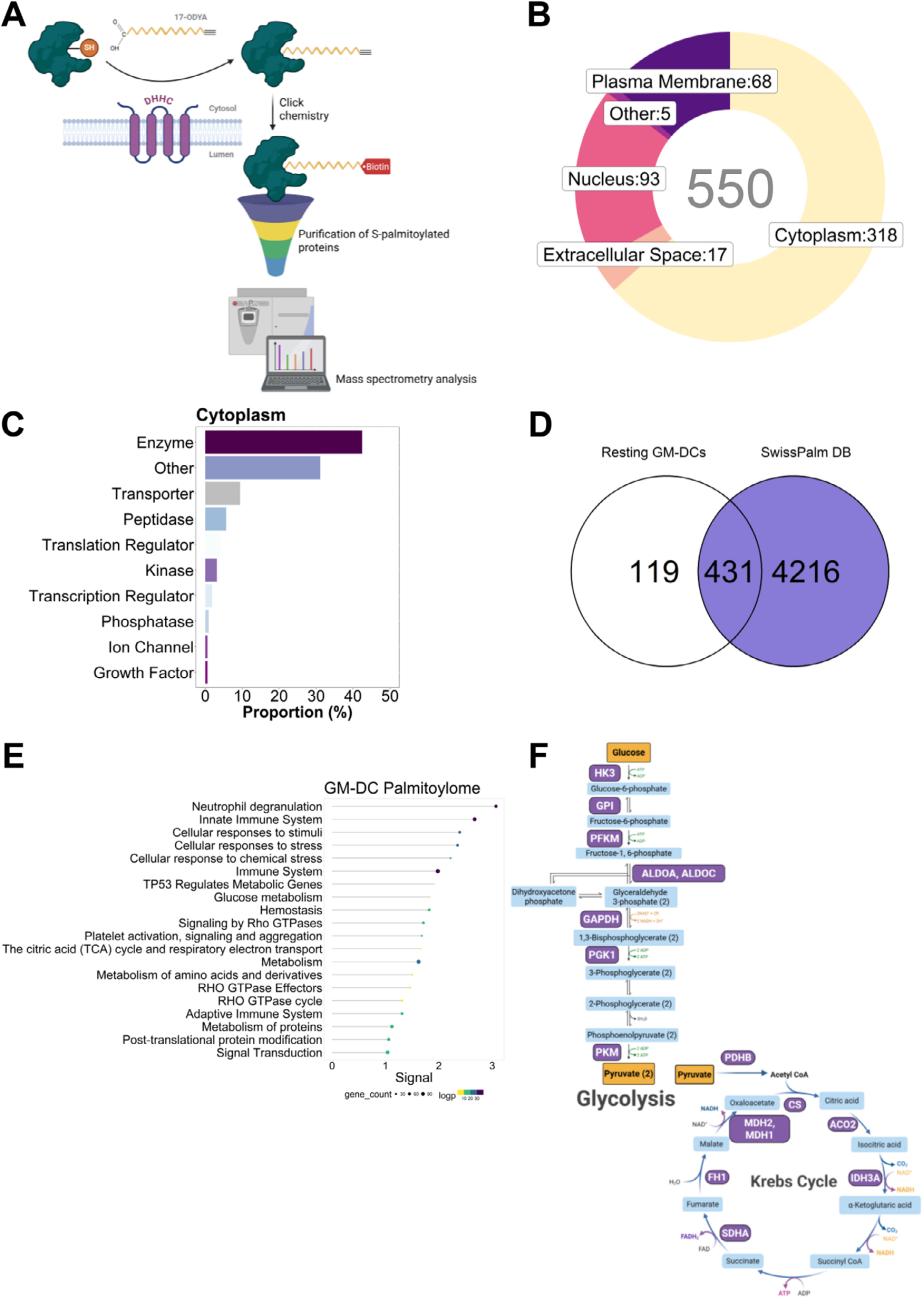
Palmitoyl‐proteome of homeostatic GM‐DCs. Resting bulk GM‐CSF cultures were generated from the bone marrow of C57BL/6 mice. Cells were then incubated with 17‐ODYA or vehicle (DMSO) for 4 h, followed by protein extraction and processing for click chemistry‐based enrichment of S‐palmitoylated proteins and MS identification. (A) Schematic overview of the MS–based workflow used to identify S‐palmitoylated proteins in GM‐DCs, incorporating metabolic labeling with 17‐ODYA and click chemistry–based enrichment strategies. (B) Donut chart showing the number of lipid‐modified proteins grouped by subcellular localization, as annotated by IPA. (C) Proportion of cytoplasmic proteins separated by their functional annotation. (D) Venn diagram comparing the S‐palmitoylated proteins identified in this study with curated entries from the SwissPalm database. (E) Pathway enrichment analysis of the identified S‐palmitoylated proteins (17‐ODYA/DMSO), displaying the top 20 overrepresented pathways. Each category displays the number of associated protein‐coding genes, and their statistical significance represented as −log (*p*‐value). (F) Diagram of glycolysis and the Krebs cycle reactions highlighting enzymes identified as S‐palmitoylated targets in this study (indicated in purple). Three independent samples (*n* = 3) were evaluated in a single experiment, including three technical replicates. The dataset generated after raw data processing (see Methods) was subjected to downstream analysis based on proteins meeting the following criteria: |log_2_FC| > 1 and FDR < 0.01.

To explore the dynamic nature of S‐palmitoylation in resting GM‐DCs, we first assessed the kinetics of Palm‐FITC incorporation, aiming to capture a broad spectrum of S‐palmitoylated targets. Our findings indicated that the peak incorporation occurred 4 h following probe exposure (data not shown). Subsequently, we conducted large‐scale profiling of S‐palmitoylated proteins by introducing 17‐ODYA into GM‐DC cultures for 4 h. This palmitic acid analog contains an alkyne moiety, enabling subsequent Cu⁺‐catalyzed cycloaddition (“click chemistry”) for biotin conjugation and is recognized as a substrate by PAT, and covalently linked to cysteine residues [23]. Following click‐chemistry‐based biotinylation, the biotin‐tagged proteins were further purified and characterized by MS (Figure 1A). Only proteins that met the filtering threshold were included in the subsequent analysis (see Methods).

Our results demonstrated that resting GM‐DCs exhibited a diverse array of 550 S‐palmitoylated proteins (Table S1). We applied fold‐change‐based classification criteria like those used in previous studies [24] and found that 50% of the identified proteins met high or medium confidence thresholds (Table S2). Since PATs are integral membrane proteins, it is expected that most S‐palmitoylated targets will be localized to cellular membranes. Nevertheless, based on the Ingenuity Pathway Analysis (IPA) analysis [25], our set of 550 S‐palmitoylated proteins was predicted to be distributed across distinct subcellular compartments (Figure 1B). Notably, most of the cytoplasmic proteins demonstrated enzymatic activity (Figure 1C). To validate our findings, we compared our dataset with an open database for protein S‐palmitoylation, SwissPalm [26]. We found that 78% of the proteins in our dataset were previously reported as S‐palmitoylated targets in distinct experimental settings (Figure 1D), confirming the specificity of our workflow. Additionally, several of these proteins were annotated as palmitate‐modified targets according to the UniProtKB classification system (KW‐0564). This includes well‐characterized S‐palmitoylated proteins such as HRAS, NRAS, CANX, CD36, and several ribosomal proteins (Figure S1A). Further pathway enrichment analysis revealed overrepresented signatures associated with immune response and cell metabolism (Figure 1E). The latter includes key enzymes related to glycolysis (HK3, GPI, PFKP, ALDOC, ALDOA, GAPDH, PGK1, PKM) and the Krebs cycle (PDHB, CS, ACO2, IDH3A, SDHA, FH1, MDH1, MDH2) (Figure 1F). On the other hand, several S‐palmitoylated targets that did not match SwissPalm entries (119 out of 550) were associated with innate immunity and inflammasome signaling (Figure S1B). This subset contained well‐known DC markers, such as ITGAX (CD11c), along with antigen‐presenting molecules (H2‐DMA and H2‐AA), and inflammasome‐related proteins, including ASC (encoded by *Pycard*) and CASP8. Moreover, the STRING [27] interaction enrichment analysis indicated that these proteins are functionally connected, highlighting strong associations between CASP8, ASC, and TIPE2 (encoded by *Tnfaip8l2*) based on experimental evidence (Table S3). Although these proteins require experimental validation as potential S‐palmitoylation targets, the GPS‐Palm [28] algorithm confidently predicted multiple cysteine hotspots on CASP8, along with additional putative palmitoylation sites on ASC and TIPE2 (Figure S1C).

Notably, our results reveal that resting GM‐DCs rely on the S‐palmitoylation of crucial proteins involved in immune responses and metabolic pathways, highlighting S‐palmitoylation as a potential regulatory mechanism at the steady state.

### 2.2 The Repertoire of S‐Palmitoylated Proteins Undergoes Significant Remodeling Upon TLR9 Activation

DCs are equipped with DNA sensors that play a crucial role in calibrating immune responses against cancer and viral infections [29]. This includes a subset of cytoplasmic sensors such as cGAS‐STING and AIM2, as well as endosome‐restricted receptors like TLR9. Almost all DC subsets express TLR9, and upon ligand activation, they respond by secreting proinflammatory cytokines, producing type I interferons, and priming T cells [30]. In this context, S‐palmitoylation of key proteins involved in TLR signaling and interferon‐related pathways has been reported in macrophages and DCs [31]. Nevertheless, there have been no studies linking TLR9‐mediated DC activation to the remodeling of the S‐palmitoylated proteome. Therefore, we conducted a comparative analysis of S‐palmitoylated proteins between resting GM‐DC and following TLR9 activation with CpGB.

**FIGURE 2:**
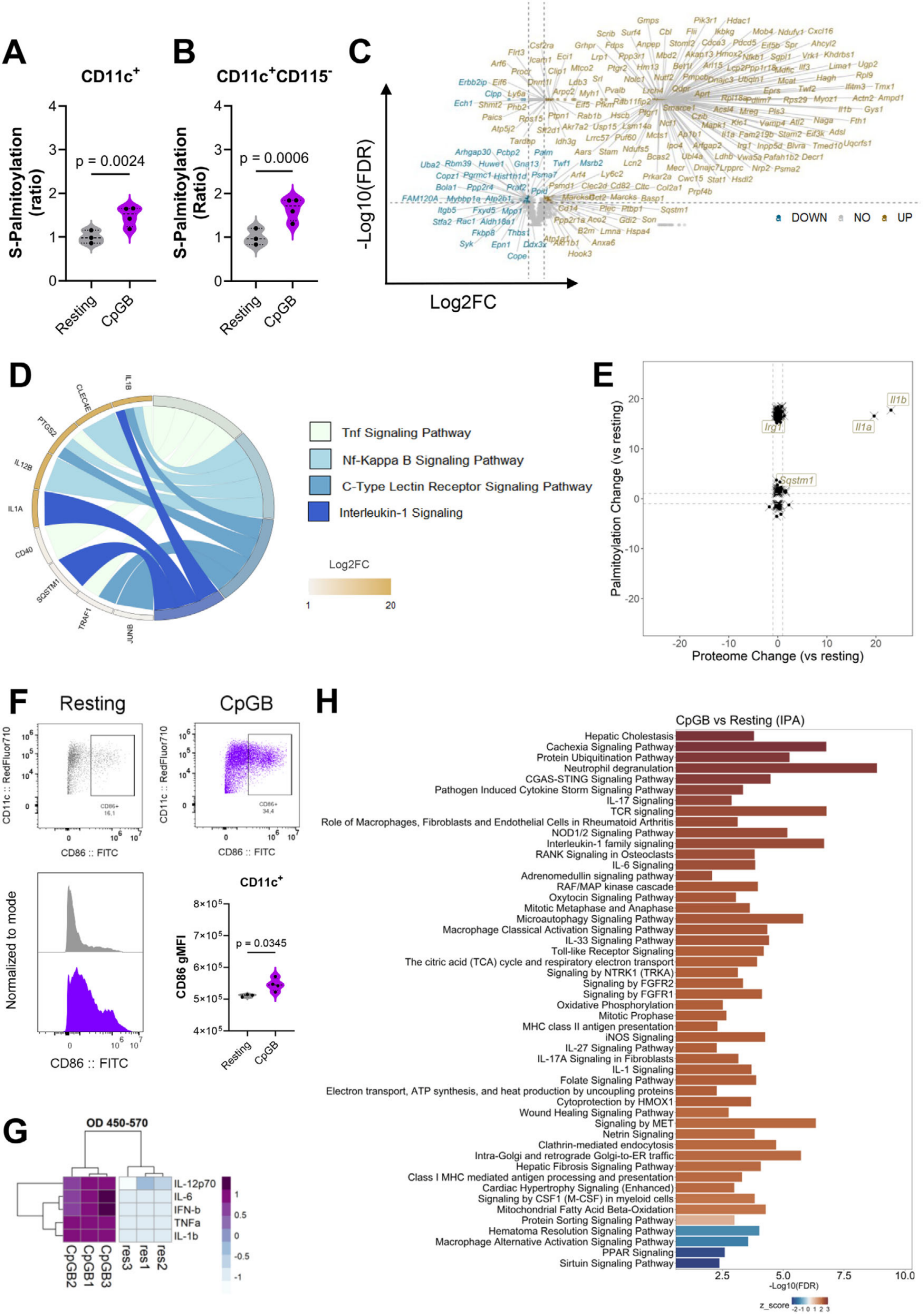
TLR9 activation prompts S‐palmitoylated proteome remodeling. Bulk GM‐CSF‐derived cultures were stimulated with 1 µM CpGB for 4 h or left unstimulated (resting) in the presence of 17‐ODYA, and subject to S‐palmitoylated proteome analysis using click‐MS. In parallel, resting and CpGB–activated cultures were processed for whole proteome profiling via MS. Relative S‐palmitoylation levels in (A) total CD11c⁺ cells and (B) DC‐like populations from CpGB‐stimulated GM‐DC cultures, compared with resting. (C) Volcano plot showing S‐palmitoylated proteins in CpGB‐stimulated GM‐DCs compared with resting controls. Proteins that met the statistical threshold are labeled based on their coding genes. (D) Chord diagram illustrating enriched biological pathways at the whole‐proteome level in GM‐DCs following TLR9 activation, compared with the resting condition (CpGB/resting). Protein‐coding genes arranged by log_2_FC are linked with their signaling pathways. (E) Scatter plot showing the relationship between whole‐proteome protein abundance changes and S‐palmitoylation enrichment in CpGB‐stimulated GM‐DCs. Each point represents a protein, with the x‐axis indicating log_2_FC in total protein abundance (CpGB/resting) and the y‐axis indicating log_2_FC change in S‐palmitoylation (17‐ODYA CpGB/resting). Proteins displaying a simultaneous increase in protein abundance and S‐palmitoylation are labeled. Dash line indicated threshold of |log_2_FC|>2. (F, G) Evaluation of activation markers and cytokine secretion in resting and TLR9‐stimulated GM‐Ds. CD86 expression was assessed in CD11c⁺ cells by FACS. Cytokine levels were measured and displayed as a heatmap using scaled ΔOD450‐570 values. (H) Pathway enrichment analysis performed on S‐palmitoylated proteins that were differentially abundant between CpGB–stimulated and resting GM‐DC cultures. For the S‐palmitoylated proteome analysis, three independent biological replicates (*n* = 3) were evaluated in a single run, each analyzed in technical triplicate. Regarding the whole proteome, all samples (*n* = 4) were processed in technical duplicates. The dataset generated after raw data processing (see Methods) was subjected to downstream analysis based on proteins meeting the following criteria: |log_2_FC| > 1 and FDR < 0.01. Experiments involving DC maturation included 3–4 biological replicates, assessed in three independent runs. Data are presented as mean ± SD. Unpaired parametric *t*‐tests were used to compare resting and CpGB–stimulated DCs. Statistically significant differences were defined as *p*‐value < 0.05 and are indicated accordingly.

A 4 h CpGB stimulation resulted in a relative increase in S‐palmitoylation levels in total CD11c⁺ cells and the CD11c⁺CD115− DC population (Figure 2A,B), without affecting the uptake of exogenous FAs (Figure S2A). Remarkably, the S‐palmitoylated proteome of GM‐DC undergoes significant changes after TLR9 activation. Differential abundance analysis identified 185 overrepresented and 35 underrepresented S‐palmitoylated proteins in CpGB‐stimulated GM‐DCs compared with unstimulated controls (Figure 2C). We also evaluated the complete proteome of both resting and activated GM‐DC to account for potential biases in protein abundance caused by cell activation. As expected, TLR9 stimulation increased the protein expression of targets associated with NF‐κB and cytokine signaling pathways (Figure 2D). Yet, most S‐palmitoylated proteins displayed stable protein levels during CpGB stimulation (Figure 2E). Notable exceptions included interleukin‐1 beta (IL‐1β), interleukin‐1 alpha (IL‐1α), cis‐aconitate decarboxylase (CAD, encoded by *Irg1*), and sequestosome‐1 (SQSTM1), which exhibited increased S‐palmitoylation levels and were readily detectable under CpGB stimulation (Figure S2B). We observed similar outcomes in pilot MS experiments, identifying enhanced S‐palmitoylation of SQSTM1 in DCs upon TLR4 activation. SQSTM1 mediates autophagosome formation in DCs, thereby prompting antigen presentation to CD4^+^ T cells [32]. On the other hand, CAD converts cis‐aconitate into itaconate, a novel immune‐modulating molecule with antimicrobial activity [33]. Together, these signatures highlight the initiation of successful DC activation. Likewise, DC maturation was further confirmed through phenotyping, where we observed increased expression of CD86 (Figure 2F), as well as higher levels of IFN‐β, IL‐6, TNF‐α, IL‐12p70, and IL‐1β secretion after 4 h CpGB stimulation (Figure 2G).

Based on the dataset of S‐palmitoylated proteins identified as differentially abundant between CpGB‐stimulated and resting GM‐DCs (Table S4), we next executed a pathway enrichment analysis, revealing that most of the overrepresented S‐palmitoylated proteins in CpGB‐stimulated cultures were associated with immune response, including c‐GAS‐STING axis, TLR activation, and cytokine signaling (Figure 2H). Key mediators in these pathways included MAPK1, NF‐kB essential modulator (NEMO, encoded by *Ikbkg*), NF‐κB p105/p50 (NF‐κB1), signal transducer and activator of transcription‐1 (STAT1), intercellular adhesion molecule 1 (ICAM‐1), tumor necrosis factor ligand superfamily member‐15 (encoded by *Tnfsf15*), phosphatidylinositol 3‐kinase regulatory subunit‐alpha (encoded by *Pik3r1*), and C‐X‐C motif chemokine 16 (CXCL16). Regarding cell metabolism, the enrichment profiling also indicates that mitochondria‐related processes, such as metabolic transport, oxidative phosphorylation, and the TCA cycle, were overrepresented during CpGB stimulation (Figure 2H). In this setting, mitochondrial malonyl‐CoA‐acyl carrier protein transacylase (encoded by *Mcat*) and core components of the mitochondrial respiratory chain, including complex I (encoded by *Ndufv1*), ubiquinol‐cytochrome c oxidoreductase (encoded by *Uqcrfs1*), and cytochrome c oxidase (encoded by *Mtco2* and *Cox7a2*), were identified. In contrast, the activation of TLR9 signaling in GM‐DCs negatively impacts the S‐palmitoylation of proteins involved in other PTMs, such as E3 ubiquitin‐protein ligase HUWE1 (HUWE1), tyrosine‐protein kinase SYK (SYK), serine/threonine‐protein phosphatase 2A activator (encoded by *Ppp2r4*), and SUMO‐activating enzyme subunit 2 (encoded by *Uba2*) (Table S4).

Consulting the SwissPalm database revealed that 145 out of the 220 S‐palmitoylated targets identified in our dataset (66%) had been previously reported in other experimental models (Figure S2C). This subset contains a well‐known antiviral mediator, IFITM3, as well as MAPK1, STAT1, SQSTM1, and CAD. Furthermore, we have identified 75 newly S‐palmitoylated proteins that were differentially regulated within GM‐DC during TLR9 activation. This group comprised elements associated with protein dynamics, intracellular organization, and NF‐κB signaling (Figure S2D), including NF‐κB1, NEMO, ICAM1, IL‐1α, and IL‐1β. Moreover, the GPS algorithm was able to predict S‐palmitoylated sites for NF‐κB1, NEMO, IL‐1α, and IL‐1β (Figure S2E).

In summary, these findings demonstrate that TLR9 activation regulates the S‐palmitoylation of several proteins in DCs, with potential implications for crucial cellular processes including metabolism, protein dynamics, and immune response.

### 2.3 Uncovering Specific PAT Gene Expression Patterns Within DCs

Previously thought to be a spontaneous modification driven solely by local palmitic acid availability, protein S‐palmitoylation is now understood to be regulated by the action of PATs. Mammalian genomes encoded at least twenty‐three PATs, including ZDHHC1–ZDHHC9 and ZDHHC11–ZDHHC24 [34]. To gain insights into gene expression patterns of PATs and regulation in DCs, we analyzed transcriptomic data from both mouse‐ and human‐derived DCs, focusing on genes encoding ZDHHC proteins (*Zdhhc1* through *Zdhhc24*). This analysis was conducted using datasets retrieved from the Gene Expression Omnibus (GEO) database.

**FIGURE 3:**
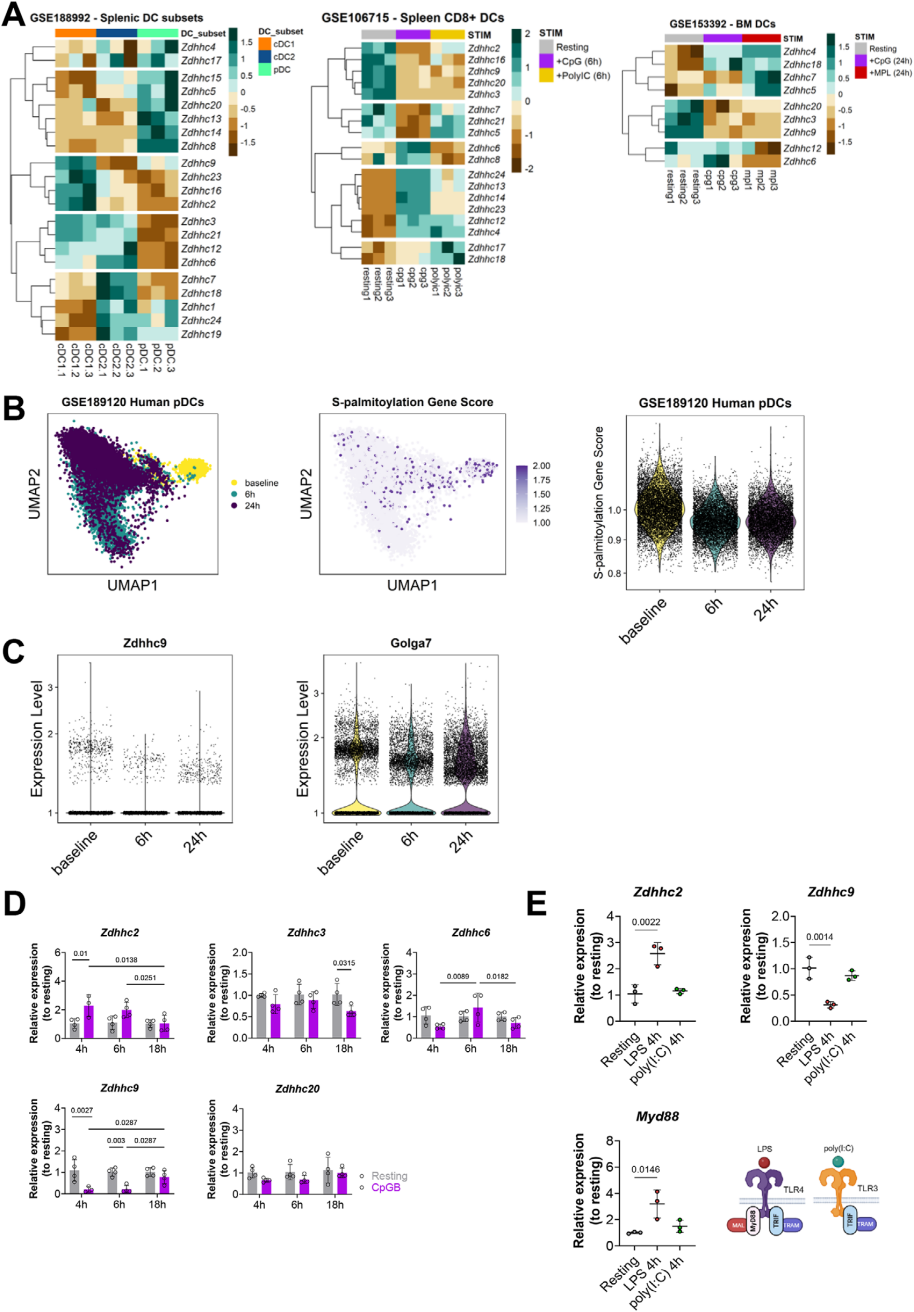
The expression of PAT‐encoding genes is differentially regulated during DC responses. Exploratory analysis of *Zdhhc* gene expression in mouse and human DC datasets, along with mRNA expression dynamics in resting and TLR‐stimulated GM‐DC cultures. (A) Heatmaps displaying DESeq2‐normalized and scaled transcript counts from two independent datasets: splenic (GSE188992 and GSE106715) and bone marrow–derived DCs (GSE153392). (B) UMAP plots generated from a single‐cell RNA‐seq dataset of human peripheral blood pDCs (GSE189120) stimulated in vitro with the influenza virus for 6 or 24 h. S‐palmitoylation gene score was calculated as detailed in Methods. (C) Violin plots showing the expression of *Zdhhc9* and *Golga7* at single‐cell resolution from human pDCs. (D) RT‐qPCR analysis of *Zdhhc2*, *Zdhhc3*, *Zdhhc6*, *Zdhhc9*, and *Zdhhc20* mRNA expression in resting and 1 µM CpGB‐stimulated GM‐DCs at 4, 6, and 18 h. Expression levels are shown relative to resting control and standardized to the housekeeping gene. (E) Gene expression levels of *Zdhhc2*, *Zdhhc9*, and *Myd88* in GM‐DC cultures upon 4 h stimulation with 100 ng/mL LPS or 50 µg/mL Poly(I:C). Representative data from three independent experiments, including biological replicates (*n* = 3–4). Data are presented as mean ± SD. One‐way or two‐way ANOVA was used to assess the effects of cell activation or stimulation duration, as appropriate. Benjamini–Hochberg post hoc analysis was applied to determine differences between groups, and *q*‐values below 0.05 were considered statistically significant and are reported accordingly.

Transcriptomic analysis of sorted DCs from the mouse spleen revealed distinct *Zdhhc* gene expression patterns across cDCs and pDCs (GSE188992) (Figure 3A). Furthermore, we noted that unstimulated DCs, whether derived from the spleen (GSE106715) or differentiated from bone marrow (GSE153392), consistently displayed higher transcript levels of *Zdhhc3*, *Zdhhc9*, and *Zdhhc20* compared with other ZDHHC‐encoding genes. However, following TLR activation, the expression levels of these genes were suppressed (Figure 3A). In addition, human‐derived pDCs that were exposed to the influenza virus displayed a single‐cell profiling characterized by reduced expression of S‐palmitoylation‐related genes compared with the baseline (Figure 3B). This included *Zdhhc9* and its accessory partner, *Golga7* (Figure 3C). To further complement these findings, we analyzed the gene expression kinetics of selected PATs in CpGB‐stimulated GM‐DCs at 4, 6, and 18 h. After 4 h of stimulation, only *Zdhhc2* exhibited a twofold increase in gene expression compared with resting GM‐DCs. At the same time, *Zdhhc3*, *Zdhhc6*, and *Zdhhc20* showed a tendency toward reduced mRNA levels at this time point, with *Zdhhc9* displaying a significant decrease (Figure 3D). Upregulation of *Zdhhc2* expression was also observed in GM‐DCs following LPS stimulation, but not upon TLR3 activation with poly(I:C), suggesting that the expression of this PAT is regulated in a MyD88‐dependent manner (Figure 3E). However, prolonged exposure to CpGB for 6 and 18 h significantly impacts the gene expression of *Zdhhc2*, *Zdhhc6*, and *Zdhhc9* (Figure 3D).

Based on these results, it is noteworthy that the expression of PAT‐encoding genes in DCs is regulated in a context‐dependent manner, involving modulation through TLR activation. In addition, transcriptomic analysis revealed that PAT gene expression is predominantly repressed upon sensing of viral ligands.

### 2.4 ZDHHC9 Shapes the Repertoire of S‐Palmitoylated Proteins During TLR9 Activation in DCs

Although the combined activity of PATs may shape the repertoire of S‐palmitoylated proteins, transcriptomic data led us to hypothesize that specific ZDHHC proteins could play a more prominent role than others in shaping the S‐palmitoylated proteome of DCs. We found that resting DCs express high levels of *Zdhhc9*, which is consistently downregulated upon CpGB stimulation in GM‐DCs and following influenza exposure in human pDCs. The activity of ZDHHC9 depends on its association with GOLGA7, which enhances its stability [20]. Notably, HRAS and NRAS have been identified as substrates of ZDHHC9 [35]. To dissect the contribution of ZDHHC9, we compared the repertoire of S‐palmitoylated proteins in GM‐DCs derived from *Zdhhc9* ^KO^ and wild‐type (WT) mice.

**FIGURE 4:**
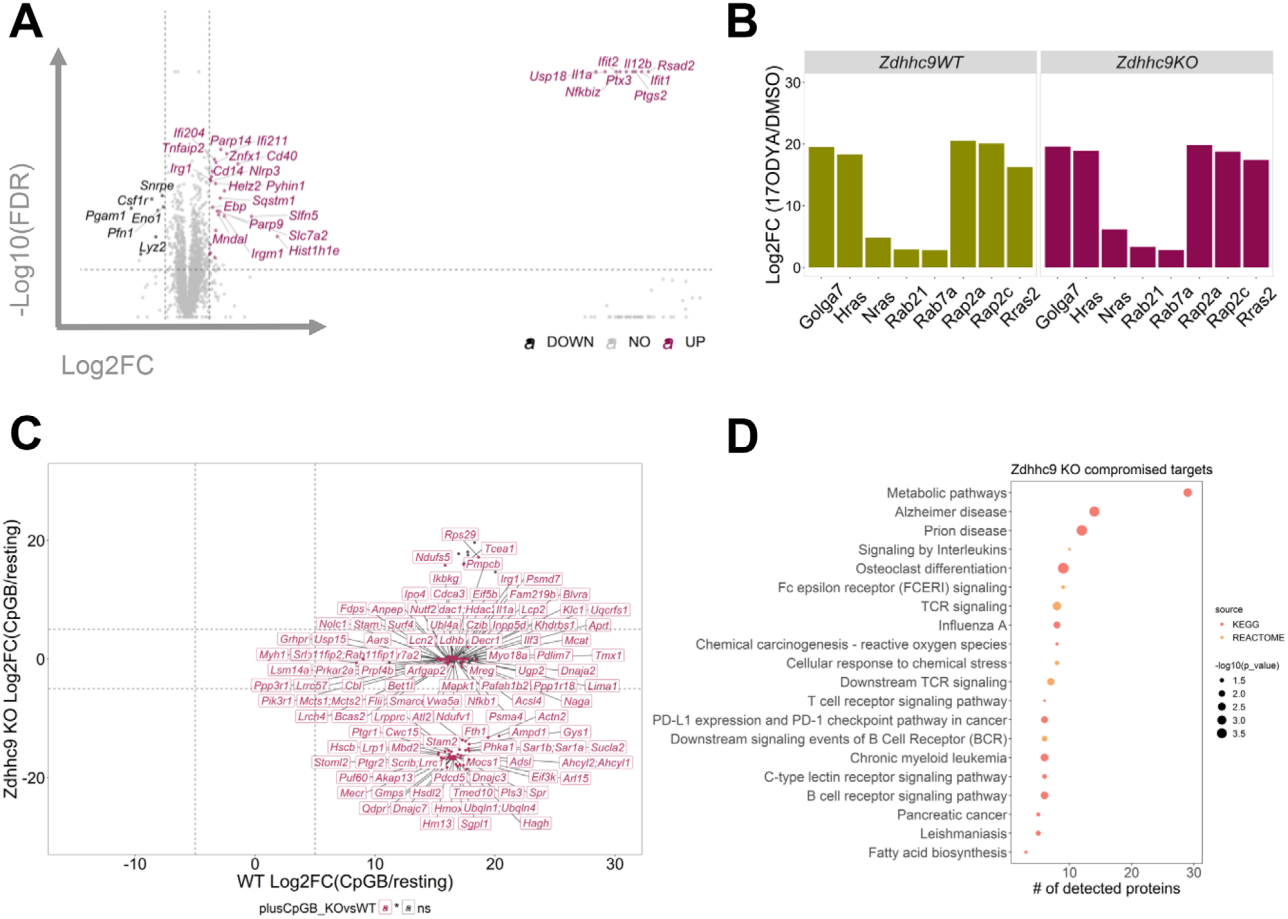
*Zdhhc9*‐deficiency compromises the S‐palmitoylation of several targets during TLR9 activation. Bulk GM‐CSF‐derived cultures isolated from *Zdhhc9* ^KO^ or WT mice were stimulated with 1 µM CpGB for 4 h or left unstimulated (resting) in the presence of 17‐ODYA or DMSO. Proteins were then extracted and subjected to S‐palmitoylated proteome profiling using Click‐MS. In parallel, whole proteome analysis was performed on resting and CpGB–stimulated GM‐DCs and compared between genotypes. (A) Volcano plot depicting the whole proteome between CpGB versus resting GM‐DCs from *Zdhhc9* ^KO^ mice. Proteins exceeding the log_2_FC and FDR thresholds are labeled (|log_2_FC| > 1 and FDR < 0.01). (B) Basal S‐palmitoylation levels of ZDHHC9‐related substrates across WT and *Zdhhc9* ^KO^ GM‐DCs. (C) Four‐way plot highlighting the S‐palmitoylated proteins found to be differentially abundant in CpGB‐activated GM‐DCs (log_2_FC > 5) contrasted by genotypes. Proteins are labeled based on their coding genes when significant differences between *Zdhhc9* ^KO^ and WT were found (CpGB KO/WT). The targets displayed in the center right and lower right panels were identified as substrates affected by *Zdhhc9* deficiency. (D) Biological process related to affected protein targets in *Zdhhc9* ^KO^ GM‐DCs. For the S‐palmitoylated proteome analysis, three independent biological replicates (WT: *n* = 3; *Zdhhc9* ^KO^: *n* = 3) were evaluated in a single run, each analyzed in technical triplicates. Regarding whole proteome, all samples (WT: *n* = 4; *Zdhhc9* ^KO^: *n* = 3) were processed in technical duplicates. The dataset generated after raw data processing (see Methods) was subjected to downstream analysis based on proteins meeting the following criteria: |log_2_FC| > 1 and FDR < 0.01.

To note, genetic deletion of *Zdhhc9* did not affect GM‐DC differentiation or CpGB‐mediated maturation *in vitro*, as reflected by the proportions of CD11c^+^ and CD86^hi^MHC‐II^+^ cells (Figure S3A,B). Furthermore, TLR9 stimulation elicited global proteomic changes that closely resembled those observed in WT GM‐DCs (Figure 4A). Unexpectedly, the S‐palmitoylation levels of HRAS, NRAS, GOLGA7, as well as several Ras‐related proteins, remained unaffected in resting *Zdhhc9* ^KO^ GM‐DCs (Figure 4B). In contrast, during TLR9 activation, the four‐way plot revealed genotype‐dependent modulation of S‐palmitoylated targets after 4 h of CpGB stimulation (Figure 4C). This enabled us to classify the dataset into two well‐defined groups of S‐palmitoylated targets: those unaffected (upper right quadrant) and TLR9‐regulated proteins with compromised S‐palmitoylation due to *Zdhhc9* depletion (middle and lower right quadrants; Table S5). Among these were previously reported ZDHHC9 substrates, including lactate dehydrogenase (encoded by *Ldhb*) [36]. Further enrichment analysis of this protein set revealed significant overrepresentation of immune and metabolic‐related processes (Figure 4D), including members of the TCA cycle (*Ndufv1*, *Cox7a2*, and *Uqcrfs1*) and glycogen biosynthesis (*Gys1* and *Ugp2*). Accordingly, the TLR9‐dependent increase in S‐palmitoylation of NF‐κB axis components, the antiviral mediator IFITM3, the itaconate‐producing enzyme CAD, and glycogen metabolism‐associated proteins were impaired in *Zdhhc9* ^KO^ GM‐DCs.

Our data demonstrated that ZDHHC9 activity contributed to shaping the repertoire of S‐palmitoylated proteins during TLR9 activation in DCs, uncovering potential ZDHHC9‐restricted substrates linked to immune response and cell metabolism.

### 2.5 Zdhhc9 expression Is Dispensable for the Proper Differentiation and Maturation of DCs

Growing evidence on S‐palmitoylation highlights a strong link between PAT activity and the regulation of immune responses. Since our findings demonstrated that ZDHHC9 activity influences the modulation of S‐palmitoylation in immune‐related proteins, we questioned whether genetic deletion of *Zdhhc9* could affect DC function. To answer this, we investigated key aspects of DC biology, including maturation, cytokine production, and T cell priming.

**FIGURE 5:**
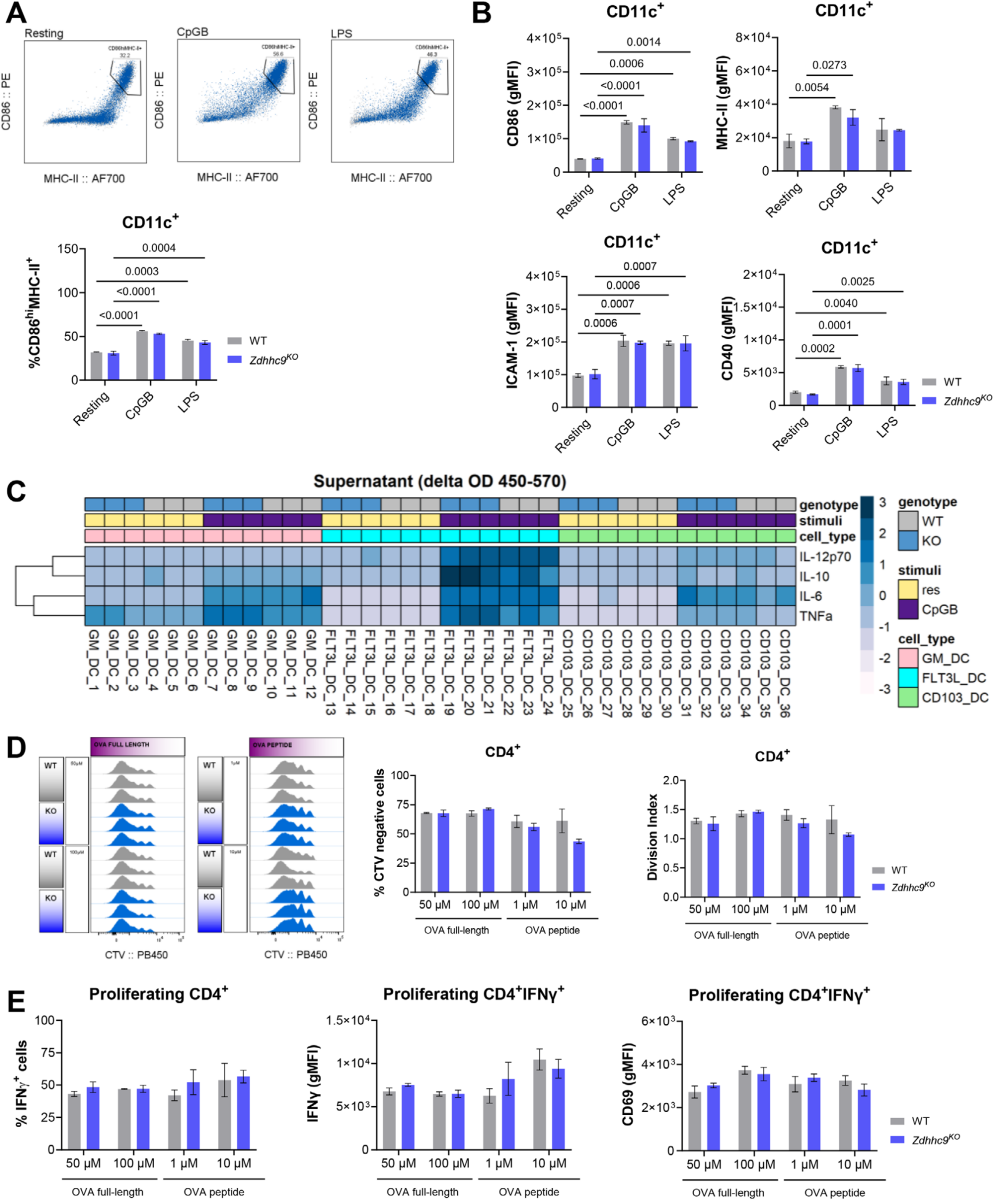
*Zdhhc9* deficiency does not impact the functional properties of DCs. DC cultures generated from *Zdhhc9* ^KO^ or WT mice were stimulated with 1 µM CpGB or 100 ng/mL LPS for 4 h, or left unstimulated (resting). Markers of DC maturation and T cell priming capacity were evaluated and compared between genotypes. (A) TLR‐driven maturation of GM‐DCs evaluated as frequency (%) of CD86^hi^MHC‐II^+^ cells gate in CD11c^+^. Representative dot plots for each condition are displayed. (B) Bar plots depicting gMFI for co‐stimulatory (CD86 and CD40) and activation markers (MHC‐II and ICAM‐1) within resting CD11c^+^ GM‐DCs. (C) Heatmap displaying scaled ΔOD_450‐570_ values for IL‐12p70, IL‐10, IL‐6, and TNFα, from culture supernatant. Cytokine levels were measured in resting and 4 h CpGB–stimulated GM‐DC, FLT3L‐DC, and CD103⁺ DC cultures, and compared between genotypes. (D) Proliferation of OTI‐II CD4^+^ T cells following a 4‐day co‐culture with Flt3L‐DCs previously loaded with varying concentrations of OVA‐FL or OVA peptide in the presence of 1 µM CpGB. Proliferation was assessed by CTV staining on CD4^+^ T cells, as shown in the histograms. The % of CTV‐negative cells and division index within CD4^+^ were calculated in FlowJo. (E) The % of IFN‐γ+ cells and protein expression levels for IFN‐γ and CD69 evaluated in proliferating CD4^+^ T cells (CTV negative). Results include biological replicates (WT: *n* = 3; *Zdhhc9* ^KO^: *n* = 3) and are representative of three independent experiments. Data are presented as mean ± SD. Statistical analysis was performed using two‐way ANOVA followed by multiple comparisons with FDR correction (significance threshold: *q*‐value < 0.05). Only statistically significant differences are shown and reported as q‐values in the figure.

**FIGURE 6:**
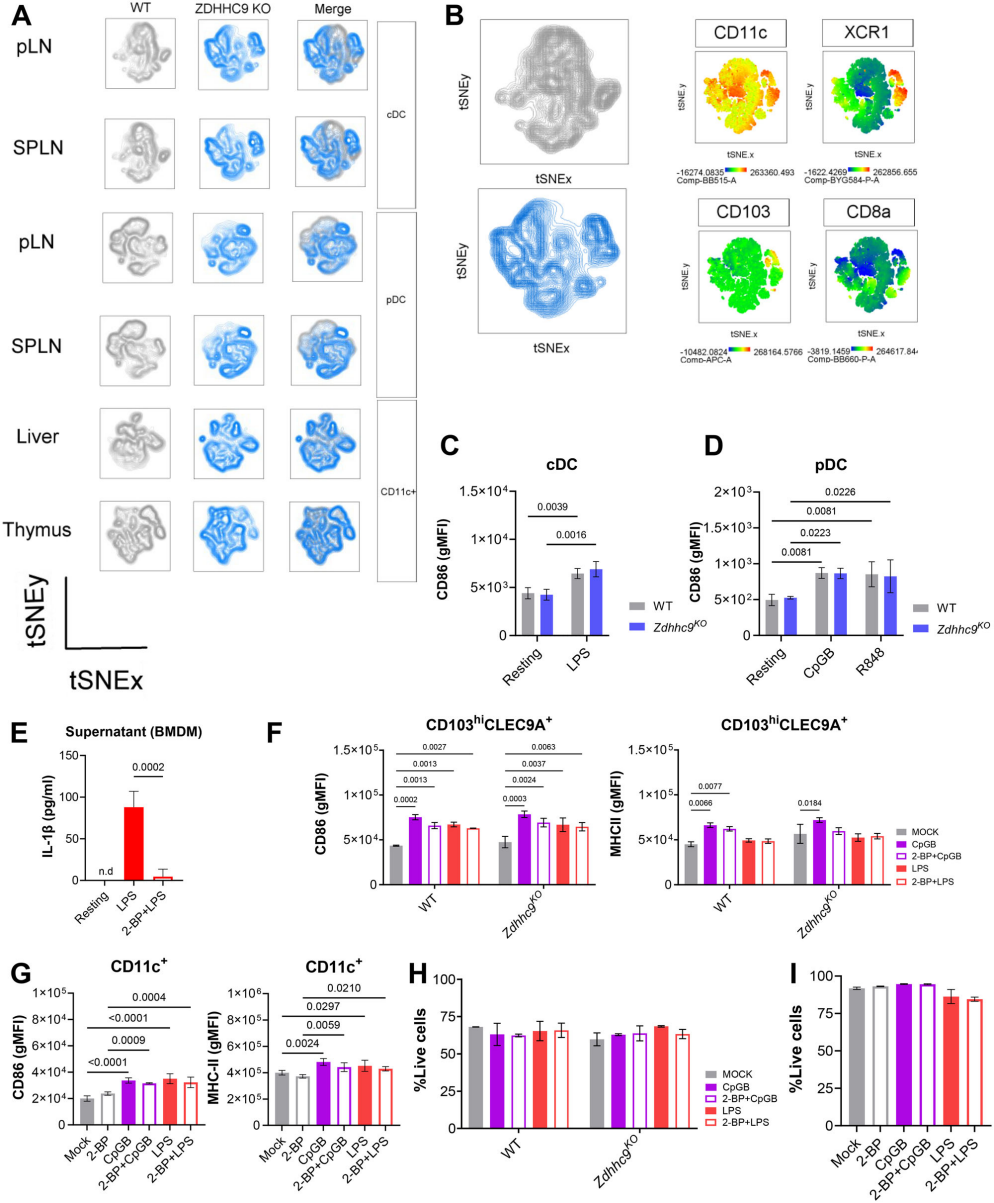
General inhibition of PATs with 2‐BP or specific depletion of *Zdhhc9* does not affect DC differentiation or their responsiveness to TLR stimulation. Phenotypic and functional characterization of tissue DC subsets derived from *Zdhhc9* ^KO^ or WT mice, and evaluation of *in vitro* DC maturation following global S‐palmitoylation inhibition with 2‐BP. (A) t‐SNE plots were generated from FACS data to visualize the genotype‐dependent phenotypic differences of cDCs and pDCs derived from the SPLN and pLNs, as well as CD11c⁺ cells isolated from the liver and Thy. The compensate parameters considered for dimensionality reduction were selected as follows: cDCs (CD11c, CD11b, XCR1, CD103 and, CD8a), pDCs (CD11c, CD11b, CD9, Sca‐1, Ly6D, PDCA‐1, CCR9), liver CD11c^+^ (CD11c, CD11b, SIGLECH, PDCA‐1, MHC‐II, CD103), and Thy‐derived CD11c^+^ (CD11c, B220, CD8a, PDCA‐1, CD103, and SIRP‐1a). More details about data processing are provided in Methods. (B) tSNE plot with expression levels for CD11c, XCR1, CD103 and CD8a within cDC subsets. (C, D) CD86 expression on cDC (CD11c^+^B220^−^MHC‐II^hi^) and pDC (CD11c^+^B220^+^PDCA‐1^+^) derived from *ex vivo* splenocyte cultures stimulated with TLR agonists (1 µM CpGB, 100 ng/ml LPS, or 3 µM R848). (E) BMMs were preincubated with 100 µM 2‐BP for 2 h and later stimulated with 100 ng/mL LPS for 4 h. The secretion of IL‐1β was induced with 1 mM ATP for 45 min and measured in the culture supernatant by ELISA. Undetectable cytokine levels are indicated as n.d. (not detected). (F) CD103^+^DC cultures generated from WT and *Zdhhc9* ^KO^ mice were pretreated with 100 µM 2‐BP for 2 h before stimulation with 1 µM CpGB or 100 ng/mL LPS for 6 h. The expression of CD86 and MHC‐II was inspected by FACS within CD103^hi^CLEC9A^+^ cells. (G) GM‐DC cultures exposed to 100 µM 2‐BP or vehicle for 2 h and later activated for 4 h with 1 µM CpGB or 100 ng/mL LPS. The expression of activation markers (CD86 and MHC‐II) was evaluated within CD11c^+^ cells by FACS. (H, I) Cell viability evaluated as % of LIVE DEAD AQUA negative cells by FACS. Data are supported by two independent experiments, including biological replicates (WT: *n* = 3–4; *Zdhhc9* ^KO^: *n* = 3). Data are presented as mean ± SD. One‐way or two‐way ANOVA with multiple testing correction was applied as appropriate. Significant *q*‐values are reported considering a threshold of FDR < 0.05.

The TLR‐mediated induction of DC maturation, marked by a higher frequency of CD86^hi^MHC‐II^+^ cells, was similar between GM‐DCs from *Zdhhc9* ^KO^ and WT mice (Figure 5A). Additionally, we did not observe genotype‐related differences in the protein expression of key activation markers, including costimulatory molecules CD40 and CD86, MHC‐II, or ICAM‐1 (Figure 5B). These findings were confirmed using different in vitro culture strategies, such as the well‐established Flt3L‐induced bone marrow‐derived DCs (Flt3L‐DCs) and our protocol for generating CD103⁺ DCs [21], both of which showed no impact on DC differentiation or activation due to *Zdhhc9* deletion (Figure S4A–F). Similarly, IL‐12p70, IL‐10, IL‐6, and TNFα secretion remained unaffected (Figure 5C). Furthermore, we performed co‐culture experiments with WT or *Zdhhc9* ^KO^ DCs, loaded with either full‐length OVA protein or the OVA_323–339_ peptide, and incubated with CD4⁺ T cells isolated from OT‐II mice. Changes in CD4⁺ T cell priming were assessed by evaluating proliferation, IFN‐γ production, and CD69 expression using flow cytometry (FACS). Overall, the frequency of CTV‐negative CD4⁺ T cells and division rates were comparable between genotypes, with a modest reduction observed upon stimulation with 10 µM OVA peptide (Figure 5D). However, the final induction of IFNγ‐producing CD4+ T cells and activation marker expression, such as CD69, remained unaffected (Figure 5E). A more detailed analysis of cDC and pDC subsets from peripheral lymph nodes (pLNs) and spleen (SPLN), as well as CD11c⁺ subsets isolated from liver and thymus (Thy), showed discrete differences between genotypes (Figure 6A). Notably, a cDC subset expressing XCR1^hi^CD103^hi^CD8a^low^ was enriched in both pLNs and SPLN from *Zdhhc9* ^KO^ mice (Figure 6B). However, splenic pDCs and cDCs derived from either WT or *Zdhhc9* ^KO^ mice exhibited a comparable activation, as evidenced by increased CD86 expression following TLR stimulation (Figures 6C,D).

To complement these experiments, we exposed DCs to 2‐bromopalmitate (2‐BP), a broad‐spectrum inhibitor of PATs. First, we confirmed that a 2 h preincubation with 100 µM 2‐BP before LPS stimulation in bone marrow macrophages (BMMs) was sufficient to impair IL‐1β secretion, consistent with recent reports [37](Figure 6E). Nevertheless, CpGB‐ or LPS‐induced modulation of CD86 and MHC‐II expression in CD103⁺ DCs remained unaffected by 2‐BP treatment, regardless of genotype (Figure 6F). Additional evaluations on WT GM‐DCs did not reveal further differences in maturation markers due to 2‐BP exposure (Figure 6G). In both cases, cell viability was not significantly affected (Figures 6H,I).

In summary, our results indicate that deletion of *Zdhhc9* alone does not affect DC differentiation and TLR‐induced maturation. Additionally, our data show that short‐term incubation with 2‐BP does not impair DC maturation in vitro.

## 3 Discussion

Due to their low abundance and the challenges of isolating them from tissues, investigating the S‐palmitoylated proteome in DCs relies on in vitro strategies that allow for sufficient cell yields. To date, only a few reports have described the repertoire of S‐palmitoylated proteins in the DC2.4 cell line. These pioneering studies identified S‐palmitoylated targets related to DC function, such as TLRs and IFITM3 [24, 38]. In this work, we successfully implemented large‐scale profiling of S‐palmitoylated proteins in GM‐DC cultures by combining a click chemistry‐based method with MS. The use of 17‐ODYA as a metabolic label enhanced the detection of hydroxylamine‐sensitive proteins, thereby enabling the selective identification of S‐palmitoylated targets over other lipid modifications (i.e., N‐myristoylation) [23]. We identified 550 S‐palmitoylated proteins involved in immune responses and cellular metabolism, confirming homeostatic S‐palmitoylation in GM‐DC cultures. The majority were localized in the cytoplasm, suggesting that S‐palmitoylation may regulate their interactions and membrane association. Further validation of our dataset revealed the presence of well‐known S‐palmitoylated proteins reported in experimental databases. Additionally, we identified novel targets with potential relevance for immune function, such as TIPE2 and CASP8. TIPE2 is a crucial regulator of immune homeostasis, which is preferentially expressed in lymphoid and inflamed tissues [39]. CASP8 can interact with TIPE2 through its death effector domains (DED), negatively regulating TLR signaling [40]. Remarkably, our analysis revealed putative S‐palmitoylation sites within the DED regions of both TIPE2 and CASP8, suggesting a potential role for this lipid modification in influencing their function. Overall differences in the number and nature of identified targets compared with previous datasets may be attributed not only to the cell origin but also to time‐dependent profiles influenced by 17‐ODYA incorporation, as described by others [41]. In this context, we observed the strongest S‐palmitoylation signal in GM‐DC cultures after 4 h of incubation with 17‐ODYA. Although GM‐DCs comprise a heterogeneous population of cells [42, 43], including monocyte‐like subsets that may significantly influence proteomic outcomes, they are better suited for studying immune signaling and metabolic pathways than immortalized cell lines. Therefore, in this study, we provide a robust methodological framework for future studies aimed at exploring protein palmitoylation in more defined and physiologically relevant DC subsets.

Although it has been demonstrated that S‐palmitoylation can influence downstream TLR signaling in DCs [44], the effect of TLR‐driven changes in the S‐palmitoylation profile remains largely unexplored. To our knowledge, this study is the first to characterize TLR9‐mediated remodeling of the S‐palmitoylated proteome in DCs. We found that short‐term exposure to CpGB caused significant changes in the spectrum of modified targets. This was due to dynamic shifts in S‐palmitoylation rather than an overall increase in protein expression or FA incorporation. Exceptions to this pattern included previously reported S‐palmitoylated targets like SQSTM1 and the itaconate‐producing enzyme CAD. Notably, lipidation of SQSTM1 has been suggested to play an essential role in maintaining autophagosome formation [45], whereas the effect of S‐palmitoylation on CAD activity remains poorly understood. Following CpGB stimulation, we observed a notable enrichment of S‐palmitoylated proteins involved in DNA‐sensing pathways. This supports the idea that effective TLR9 activation may rely on the S‐palmitoylation of downstream mediators. Several proteins in this dataset are listed in established S‐palmitoylation databases. Furthermore, we extended the experimental foundation by identifying novel lipid‐modified proteins linked to TLR9 activation, including members of the inflammasome signaling pathway such as NF‐κB1, NEMO, IL1α, and IL1β, with predicted S‐palmitoylation sites. Because of its ability to regulate the expression of inflammatory genes, NF‐κB activity is tightly controlled by IKK complexes and inhibitory IκB proteins [46, 47]. Canonical activation involves cooperation with the IKK complex, which includes the kinases IKKα and IKKβ, and the regulatory subunit NEMO [48]. PTMs like ubiquitination and SUMOylation of NEMO are crucial for proper IKK function [49]. Additionally, lipid modification of Cys‐62 within the p50 subunit of NF‐κB1 has been shown to inhibit its DNA‐binding ability [50]. We identified two cysteine residues within the RHD domain, known to mediate NF‐κB1–DNA interactions, that are highly susceptible to S‐palmitoylation according to algorithm predictions. However, the precise role of S‐palmitoylation in regulating downstream inflammatory pathways requires further study. Recent research has identified lipidation as a key modulator of NLRP3 inflammasome assembly [51]. Regarding IL‐1α and IL‐1β, we observed a marked increase in both protein levels and S‐palmitoylation during DC activation. This was further supported by the increased release of IL‐1β into the culture supernatant after 4 h of CpGB stimulation. Although some components of the IL‐1 secretory pathway have been reported as lipid‐modified targets [52, 53], S‐palmitoylation of IL‐1β and IL‐1α has not been previously reported. Additionally, mediators of oxidative phosphorylation and the TCA cycle were enriched in the subset of S‐palmitoylated proteins regulated by TLR9 activation. Emerging evidence supports a role for S‐palmitoylation in regulating mitochondria‐localized proteins [54]. Furthermore, S‐palmitoylation of core mitochondrial respiratory chain components like *Ndufv1* and *Mtco2* has been previously described in immune cells [38, 55]. Overall, our findings reveal a broad range of S‐palmitoylated targets involved in immune responses and cellular metabolism that are differentially regulated during TLR9‐mediated activation in DC cultures.

S‐palmitoylation is the most prevalent form of protein lipidation, likely due to the high intracellular abundance of 16‐carbon palmitoyl‐CoA [13, 56]. The enzymatic attachment of the lipid moiety to cysteine residues is catalyzed by the ZDHHC protein family, which exhibits restricted expression in eukaryotes [57]. Moreover, the evaluation of PAT‐encoding genes suggested cell‐type‐specific expression patterns in the brain [58]. Accordingly, curated RNA‐seq datasets were generated to examine the tissue‐specific expression of *Zdhhc* genes [59]. Nevertheless, expression profiling and regulation of PATs in DCs remain poorly characterized. By inspecting transcriptomic datasets, we characterized expression patterns of PAT‐encoding genes in DC subsets from both mice and humans. Furthermore, this work inspected TLR‐driven changes in gene expression, revealing differential regulation of ZDHHC gene clusters upon TLR stimulation. In particular, the expression of *Zdhhc9* was repressed during TLR9 sensing in GM‐DCs, but also when human pDCs were exposed to influenza. Modulation of *Zdhhc9* expression has been shown to directly influence PD‐L1 expression [60]. Moreover, lower mRNA levels of *Zdhhc9* were correlated with increased immune cell infiltration in tumor biopsies [61]. Together, these findings support an immunosuppressive role for ZDHHC9, which is associated with an unfavorable prognosis in cancer [62]. On the other hand, we observed upregulated *Zdhhc2* expression in DCs that was restricted to TLR9 and TLR4 signaling. Recent reports emphasized that pDCs require ZDHHC2 for IFN‐α production [63]. In addition, *Zdhhc2* deficiency in macrophages impaired immune responses against *Mycobacterium tuberculosis* [64]. Given this background, understanding the transcriptomic landscape of ZDHHC expression may offer valuable insights for prioritizing candidate genes for further investigation, especially those implicated in DC function.

This study provides further insight into the regulatory role of ZDHHC9 in modulating the S‐palmitoylated proteome during DC activation. Among all PAT‐encoding genes, *Zdhhc9* exhibited one of the highest expression levels under resting conditions. Upon CpGB stimulation, its expression significantly decreased at 4 h but was restored to baseline by 18 h, indicating a transient regulatory response. We identified protein targets whose S‐palmitoylation is either directly or indirectly regulated by *Zdhhc9* expression upon TLR9 engagement. The application of novel technologies to track PAT substrate specificities will enable the further validation of these proteins as ZDHHC9‐specific targets [65]. Moreover, experiments employing catalytically inactive ZDHHC9 mutants should be considered to directly assess the functional role of this PAT in TLR9‐mediated signaling. Interestingly, the basal S‐palmitoylation levels of NRAS and HRAS—well‐known substrates of the ZDHHC9/GOLGA7 complex—remained unchanged in *Zdhhc9*‐deficient GM‐DCs. This may be attributed to substrate redundancy, as ZDHHC14/GOLGA7 and ZDHHC18/GOLGA7 complexes have also been reported to mediate the S‐palmitoylation of NRAS and HRAS [66]. On the other hand, the S‐palmitoylation of novel targets modulated by TLR9 activation, like NF‐κB1 and NEMO, was strongly affected due to *Zdhhc9* depletion. In this context, the observed effect may be attributed to increased palmitoylation turnover, and further investigation is needed to clarify the underlying dynamics and molecular consequences of S‐palmitoylation on these key signaling proteins. This may be particularly relevant in cancer, where aberrant ZDHHC9 expression has been reported across multiple tumor types and proposed as a potential prognostic marker [62]. In this study, we did not find major differences in the core functions of DCs between WT and *Zdhhc9*‐deficient mice. This suggests the presence of compensatory mechanisms, such as substrate redundancy or nonenzymatic fatty acylation that preserve immune signaling but also advises that ZDHHC9‐mediated S‐palmitoylation may primarily contribute to immunosuppression, as previously reported [67]. In humans, loss‐of‐function alleles in *Zdhhc9* are associated with X‐linked intellectual disability (OMIM entry: 300799) [68, 69], but their impact on the immune system has not been described. Unexpectedly, 2 h preincubation with 2‐BP before TLR9 or TLR4 stimulation did not impair the ability of DCs to express maturation markers. Previous studies showing the impact of 2‐BP in DCs have demonstrated negative modulation of responses to bacterial lipoproteins by directly interfering with TLR2 S‐palmitoylation [38]. While TLR9, TLR4, and TLR2 all engage MyD88‐dependent signaling, the recruitment of MyD88 to TLR2 and TLR4 requires the bridging adaptor TIRAP [70]. Additionally, internalization of the TLR4–ligand complex into endosomes activates MyD88‐independent signaling pathways mediated by TRIF and TRAM [71]. These findings support the notion that S‐palmitoylation may play distinct regulatory roles depending on the specific TLR pathway engaged. The 100 µM concentration of 2‐BP used in this study reflects standard practice, as it has been widely adopted in previous research [38, 72, 73, 74, 75]. In this sense, most studies have employed extended preincubation periods ranging from 8 to 24 h, suggesting that prolonged treatment may be necessary to inhibit S‐palmitoylation in cells effectively. However, we caution that 2‐BP may exert off‐target effects, as even short‐term incubation has been shown to nonselectively affect proteins beyond PATs [76, 77]. These observations underscore the urgent need to develop PAT‐specific inhibitors that can help us better understand how S‐palmitoylation modulates DC responses.

Lastly, we identified several metabolism‐related proteins with impaired S‐palmitoylation in the absence of *Zdhhc9* expression. While these findings suggest a potential role for ZDHHC9 in regulating DC metabolism, a detailed investigation falls outside the scope of this study. In this context, we did not detect genotype‐dependent differences in oxygen consumption or extracellular acidification rates in either resting or CpGB‐stimulated GM‐DCs (data not shown). Recent studies have demonstrated that ZDHHC9 mediates the S‐palmitoylation of GLUT1 [78] and CD38 [79], thereby stabilizing their membrane clustering with implications for tumor progression. This further emphasizes the complex regulatory mechanisms governed by PATs, which present both challenges and opportunities as promising therapeutic targets in cancer [80] and immune‐mediated diseases [81].

### 3.1 Data Limitations and Perspectives

This study underlies some limitations. First, the use of bulk GM‐CSF‐derived DC cultures consists of a heterogeneous population, including monocyte‐like lineages that may contribute to the proteomic readouts. The large number of cells required to investigate the S‐palmitoylated proteome by MS restricts the application of this technique in tissue‐derived DCs, which are present at low abundance relative to other immune cell populations. Ongoing methodological advances are expected to enable its application to bona fide DC subsets. Second, the broad range of proteins identified in this study, spanning multiple biological processes, presents a challenge for the comprehensive validation of S‐palmitoylation across all targets. However, cross‐validation with public databases, the application of stringent thresholds based on previous studies, and the simultaneous analysis of global proteome changes improve the confidence in the identified S‐palmitoylated targets. In addition, we identified a set of novel lipid‐modified targets with potential relevance to immune modulation, providing a foundation for future studies to investigate the specific cysteine residues involved, assess the functional impact of S‐palmitoylation, and elucidate ZDHHC substrate specificities. Lastly, the specific contribution of S‐palmitoylation in vivo and its impact on human health remain an open question and a common limitation across multiple studies. Addressing this challenge will require methodological advances and the development of selective modulators of S‐palmitoylation.

## 4 Materials and Methods

### 4.1 Animal Experiments

All the mice used in this study were housed under specific pathogen‐free conditions at the Translational Animal Research Center (TARC) of the Johannes Gutenberg University (Mainz, Germany). Mice had ad libitum access to food and water and were maintained under a 12 h light/dark cycle. Experiments involving mice were designed and conducted in accordance with the German guidelines for animal care and welfare (German TierSchG) and the European‐wide Directive 2010/63/EU. Experiments were primarily conducted using male 8–12‐week‐old C57BL/6 mice. Additionally, age‐ and sex‐matched *Zdhhc9* ^KO^ mice on a C57BL/6 background, generously provided by Prof. Dr. Luke Chamberlain [82], were incorporated for specific experimental purposes. For co‐culture experiments, OT‐II mice bearing transgenic Vα2Vβ5 TCR that recognized OVA323‐339/I‐Ab were used.

### 4.2 Isolation of Mouse Organs and Preparation of Single‐Cell Suspensions

Mice were euthanized by CO_2_ inhalation. Each mouse was subjected to heart perfusion with 5 mL cold PBS. Subsequently, secondary lymphoid organs—including axillary and inguinal pLNs and the SPLN—were harvested, along with complete resection of the Thy and liver. The organs were processed individually or pooled in groups of up to three SPLN/pLNs samples when appropriate. Organs were immediately transferred into plates containing cold RPMI (Gibco, 14190‐169) supplemented with 2% fetal calf serum (FCS) (Sigma, #F7524), 1 U/mL penicillin/streptomycin (Biochrom, #A2213), and 50 µM 2‐mercaptoethanol (Gibco, #31350‐010). Liver samples were minced into small fragments and digested at 37 °C for 30 min in complete RPMI medium (10% FCS, penicillin/streptomycin, and 2‐mercaptoethanol) supplemented with 0.5 mg/mL collagenase D (Roche, #11088866001) and 0.05 mg/mL DNase I (AppliChem, #A3778) using an orbital shaker. The addition of 500 mM EDTA stopped enzymatic digestion. Isolated pLNs, SPLN, Thy, and digested liver tissues were passed through a 100 µm cell strainer (Falcon, #352360) and mechanically dissociated using a plunger. The resulting cell suspensions were collected in 50 mL tubes containing complete RPMI medium. Cells were pelleted by centrifugation at 400×*g* and 4°C for 5 min. SPLN and liver pellets were treated with red blood cell (RBC) lysis buffer for 2 min at room temperature (RT), followed by neutralization with medium. Finally, cell suspensions from all tissues were adjusted to their final concentrations with complete RPMI, based on their intended application.

### 4.3 Generation of Immature DC Cultures from Bone Marrow Precursors

Mice were euthanized, and the tibiae and femurs were removed and placed in cold PBS. Then, intact bones were sterilized by immersing in 70% ethanol for 1 min and washed with complete RPMI. Next, the bone ends were cut, and the bone marrow (BM) was flushed out with complete RPMI into a 50 mL conical tube containing a 70 µm cell strainer (Falcon, #352350). The remaining BM retained on the strainer was smashed using a plunger and collected in the tube. The cell suspension was then centrifuged, and the pellet was resuspended in RBC lysis buffer for 1 min at RT. The lysis was stopped with complete RPMI, and cells were manually counted using a Neubauer hemocytometer. Cells were grown under sterile conditions in a CO_2_ incubator (37°C, 5% CO_2_ atmosphere). Centrifugations were always performed at 400×*g* and 4°C for 5 min. Three different strategies were employed to generate bulk GM‐DC, Flt3L‐DC, and CD103^+^DC cultures, following established guidelines [21]. For GM‐DCs, 5 × 10^6^ BM cells were plated on day 0 in 10 cm petri dishes (Sarstedt, #82.1473.001) containing 10 mL complete RPMI supplemented with the proper concentration of GM‐CSF (batch dependent). On day 2, the medium was refreshed with 10 mL complete RPMI per plate. On days 4 and 6, 10 mL of the culture media was removed from each plate and centrifuged to recover cells. The cell pellet was resuspended in 10 mL of complete RPMI supplemented with GM‐CSF at the same concentration. Cells were harvested on day 7 for further use. Both Flt3L‐DC and CD103^+^ DC cultures were generated from 15 × 10^6^ BM cells with complete RPMI supplemented with Flt3L alone or Flt3L plus GM‐CSF, respectively. Flt3L‐DCs were harvested on day 8 and prepared for further applications. For CD103^+^ DC, 5 mL of complete RPMI was added on day 6. Cells were harvested on day 9 and were repeated at a density of 3 × 10^6^ cells in 10 mL of complete RPMI supplemented with Flt3L and GM‐CSF, then cultured until day 16.

### 4.4 Production of Growth Factors for in Vitro Differentiation of DCs

Conditioned media containing growth factors were produced using transformed cell lines kindly provided by Dr. Manfred Lutz (Munich, Germany). In brief, 0.5 × 10^6^ GM‐CSF‐producing myeloma cells [83] were cultured in 175 cm^2^ flasks with 150 mL of complete RPMI until 80–90% confluency. Then the whole media was harvested and centrifuged at 400×*g*, 4°C for 7 min to separate cells. Supernatant containing GM‐CSF was collected and stored at −80°C. For Fl3tL‐conditioned media, the B16‐Fl3tL cell line [84] was seeded at 0.75 × 10^6^ in a 175 cm^2^ flask with 100 mL of complete RPMI and collected at day 7 when it reached 90% confluency. Then, the cells were pelleted by centrifugation, and the supernatant was stored at −80°C. An enzyme‐linked immunosorbent assay (ELISA) was used to measure the concentrations of GM‐CSF (R&D, #DY415) and Flt3L (R&D, #MFK00) in each batch. Further batch optimization includes determining the appropriate concentrations of growth factors to achieve optimal DC differentiation and yields across the distinct culture platforms (GM‐DCs, Flt3L‐DCs, and CD103^+^ DCs).

### 4.5 DC Treatments and TLR Stimulation

Based on the experimental readouts, DCs were seeded at 0.5–1 × 10^6^ cells in distinct culture plates and maintained in a CO_2_ incubator. TLR agonist used and final concentration are listed as follows: 1 µM CpGB ODN 1826 (TLR9; InvivoGen, #Tlrl‐1826), 1 µM CpGA ODN 1585 (TLR9; InvivoGen, #Tlrl‐1585),100 ng/ml LPS (TLR4; Sigma‐Aldrich, #L3129), 50 µg/mL poly(I:C)(TLR3; InvivoGen, #Tlrl‐picw), and 3 µM R848 (TLR7/8; InvivoGen, #Tlrl‐r848). In general, incubation periods with TLR agonists were limited to 4 h, unless otherwise specified in the figure legends. 2‐BP (Sigma Aldrich, #238422), used at 100 µM, was preincubated for 2 h before TLR stimulation, incorporating a vehicle control with DMSO. Cell suspensions were prepared in complete RPMI containing growth factors at the same concentration used during differentiation. All treatments were made in complete RPMI. Cells were processed for FACS try or real‐time PCR (qPCR), and supernatants were used for cytokine measurements.

### 4.6 Splenocyte Cell Stimulation with TLR Ligands

After ex vivo isolation as previously described, total splenic cells were counted and placed at 2 × 10^6^ cells per well in a 24‐well plate with a suitable volume of complete RPMI. Then, TLR ligands were added and incubated for 6 h in the CO_2_ incubator. Cells were collected and processed for FACS.

### 4.7 Co‐Culture Experiments with DCs and TCR Transgenic T Cells

0.2 × 10^6^ Flt3L‐DCs generated from WT and *Zdhhc9* ^KO^ were placed onto 96‐well plates and incubated overnight with specified concentrations of OVA full‐length protein (InvivoGen, #vac‐stova) or peptide fragment (OVA323‐339; Innovagen, #SP‐O323‐339) in the presence of 1 µM CpGB. The day after, CD4⁺ T cells were isolated from pooled SPLN and pLNs of age‐ and sex‐matched OT‐II mice using the Dynabeads Untouched Mouse CD4 Cells Kit (Invitrogen, #11415D). In brief, cells were filtered through a 70 µm strainer under sterile conditions and further subjected to RBC lysis. Then, negative selection of CD4+ T cells was executed according to the manufacturer's instructions. After enrichment, cells were washed twice with PBS (Gibco, #14190‐169) and stained with 2.5 µM cell trace violet (CTV; Invitrogen, #C34571) at 37°C for 7 min. Finally, CTV was thoroughly washed off, and the cells were counted and resuspended in complete RPMI at 2 × 10^6^ cells/mL. The purity of enrichment was checked by FACS. The co‐culture was established by carefully removing all but 20 µL of medium from each well of the Flt3L‐DC culture plate, followed by the addition of 100 µL of CTV‐labeled T cells. Co‐culture plates were filled up to a final 200 µL with complete RPMI and kept in a CO_2_ incubator for 4 days. Positive controls for proliferation included wells precoated with 10 µg/mL anti‐CD3ε (BioCell, #BE0001‐1), followed by the addition of CTV‐stained CD4⁺ T cells and 1 µg/mL soluble anti‐CD28 (BioCell, #BE0015‐1). Before harvesting, cells were stimulated with PMA plus ionomycin (Invitrogen, #00‐4970‐03) for 2 h, and then Brefeldin A (Invitrogen, 00‐4506‐51) was added until complete for 4 h. Finally, cells were prepared for FACS.

### 4.8 FACS Protocol for Cell Cultures and Tissues

The detailed list of antibodies and reagents used in this section is listed in Table S6. In brief, 0.5–1 × 10^6^ cells were transferred into FACS tubes and carefully washed with PBS to remove medium. Then, cells were stained with viability dye in PBS for 20 min at 4°C. After washing with PBA‐E (0.25% BSA, 0.02% sodium azide, 2 mM EDTA in PBS), cells were incubated with in‐house‐made anti‐CD16/CD32 for 10 min and subsequently stained with extracellular antibody mixes for 20 min at 4°C. A slight variation was introduced for chemokine receptors (i.e., CCR9) by incubating the antibody mix at 37°C for 30 min to amplify signal detection. When it finished, cells were washed with PBA‐E and proceeded to fixation. Cells were fixed with 2% paraformaldehyde at 4°C for 15 min. Permeabilization was conducted with PBA‐S (0.5% Saponin, 0.25% BSA, 0.02% sodium azide, 2 mM EDTA in PBS) when required. Permeabilized cells were incubated with the intracellular antibody cocktails prepared in PBA‐S for 45 min at 4°C. Lastly, cells were washed and resuspended in PBA‐E. Acquisition was performed using a Northern Lights (Cytek) or CytoFLEX S (Beckman Coulter), and data were analyzed in FlowJo (v10.10.0). The gating strategy used to assess differentiation and maturation markers across DC cultures, tissue‐derived DCs, as well as T cell proliferation and activation, is summarized in Figure S5A–F. Exploratory analysis of tissue‐DCs across genotypes was conducted as follows: CD11c⁺ cells were manually gated from each organ, and a subset of 3000 events per mouse was selected using the DownSample plugin to minimize counting bias. Dimensionality reduction of the concatenated files was then performed using t‐SNE (iterations = 1000, perplexity = 30, exact KNN algorithm), and genotype‐specific differences were assessed. The evaluated parameters were different according to the tissue origin and are detailed in the figure legends.

### 4.9 FA Uptake With BODIPY

External uptake of FAs was evaluated in resting and CpGB‐activated GM‐DCs by using BODIPY C16 FL (Invitrogen, #D3821). Briefly, 1 × 10⁶ cells were harvested and washed three times with PBS to remove the FCS. BODIPY C16 FL was diluted 1:4000 in PBS and incubated with the cells for 30 min at 37 °C in a CO_2_ incubator. When finished, cells were washed and proceeded according to the FACS workflow detailed above. Negative control included cells that were not exposed to the BODIPY probe.

### 4.10 Detection of Global S‐Palmitoylation Using Click Chemistry–Based Fluorescence Labeling

The protocol described in this section is an adaptation of the Click‐iT Cell Reaction Buffer Kit manual (Invitrogen, #C10269). 1 × 10^6^ GM‐DCs were seeded into P96‐flat flat‐bottom plates and incubated for 4 h in complete RPMI containing 25 µM of palmitic acid‐azide (Invitrogen, #C10265), in the presence or absence of 1 µM CpGB. Experimental controls included cells not exposed to palmitic acid‐azide and cells incubated on ice. The latter served as a proxy for nonspecific incorporation of the metabolic label, as uptake of exogenous FAs is suppressed under these conditions. Following incubation, cells were washed by centrifugation with PBS and subjected to viability staining as previously described. Afterward, the viability probe was removed with PBS, and cells were fixed in 2% paraformaldehyde at 4 °C for 15 min. Permeabilization was performed using PBA‐S buffer for 15 min. Cells were then washed with PBS containing 3% BSA to completely remove residual PBA‐S, a critical step to prevent EDTA interference during the click reaction. Cells were transferred to FACS tubes, and the click reaction cocktail was freshly prepared and used within 15 min. Each sample was incubated with 500 µL of the cocktail in the dark for 30 min at RT. The reaction mix contained 5 µM biotin‐alkyne conjugate (Invitrogen, #B10185), which was linked to azide‐modified palmitic acid incorporated into S‐palmitoylated proteins via Cu^2^⁺‐catalyzed cycloaddition. Finally, cells were washed and stained with an antibody panel including streptavidin‐PE/Cy5 (BioLegend, #405205) to detect the biotin‐labeled S‐palmitoylated proteins. The geometric mean fluorescence intensity (gMFI) of the PE/Cy5 signal was measured in CD11c⁺ cells and normalized to resting conditions. Similarly, the kinetics of incorporation of FITC‐conjugated palmitic acid label were analyzed in resting GM‐DC cultures at the indicated time points, following the instructions of the EZClick Palmitoylated Protein Assay Kit (BioVision, #26‐K452).

### 4.11 Isolation of S‐Palmitoylated Proteins From GM‐DC Cultures for High‐Throughput Applications

Cell numbers were carefully optimized, with a total of 20 × 10⁶ GM‐DCs used per experimental condition. Three independent biological replicates were evaluated in a single experiment. GM‐DCs were placed into petri dishes at 2 × 10^6^ cells/mL, and metabolic labelling with 20 µM of 17‐ODYA was conducted for 4 h at 37°C, in the presence or absence of 1 µM CpGB. Paired samples treated with the equivalent volume of DMSO vehicle were processed alongside. Both resting and CpGB‐stimulated DCs from either WT or *Zdhhc9* ^KO^ were harvested and extensively washed with PBS. Next, cell pellets were resuspended in lysis buffer (200 mM Tris, 4% CHAPS, 1 M NaCl, 8 M urea, pH = 8) and sonicated for 5 min at 4°C (30 s on and 30 s off). Protein lysates were clarified by centrifugation at 16,200 × *g* for 15 min at 4°C. The concentration of proteins was assessed using the Pierce protein assay (Thermo Scientific, #22660). Approximately 1 mg of protein was used as input for the Click Chemistry Capture Kit (Jena Bioscience, #CLK‐1065). Protein concentrations were standardized across samples and adjusted to a final volume of 500 µL using the urea buffer provided in the kit. As input control, preclick aliquots were separated per sample (7–10 µg of protein). For each sample, 100 µL of azide‐agarose resin (Jena Bioscience, #CLK‐1038‐2) was prewashed with 900 µL of LC‐MS grade water (Carl Roth #HN43.1) by centrifugation at 100×*g* for 5 min before use. Next, the remaining 100 µL of prewashed azide beads were mixed with protein samples and the click reaction cocktail according to the datasheet recommendations. This cocktail contained a reducing component, 100 mM of CuSO4, and Cu^2^⁺‐chelating agents. The reaction was carried out overnight (ON) at RT using an orbital rotator. The following day, the reaction mixture was centrifuged at 1000×*g* for 1 min, and the supernatant was discarded. Afterwards, the resin was washed one more time with 1 mL of ultrapure water and the supernatant discarded. At this point, agarose beads capturing 17‐ODYA‐tagged proteins were incubated with 1 mL SDS wash buffer (1% SDS, 100 mM Tris, 250 mM NaCl, 5 mM EDTA, pH 8.0) supplemented with 10 mM dithiothreitol (DTT) at 70°C for 15 min. Then, samples were rested for 30 min at RT and further centrifuged at 1000×*g* for 5 min. The supernatant was discarded, and the resin was incubated with 1 mL SDS wash buffer supplemented with 40 mM iodoacetamide (IAA) at RT for 30 min in the dark. Samples were subsequently transferred into purification columns provided in the kit. The columns were washed five times with 2 mL of SDS buffer to remove nonspecifically bound proteins, followed by ten washes with 2 mL of 8 M urea in 100 mM Tris (pH 8.0), and then ten additional washes with 2 mL of 20% acetonitrile to eliminate SDS. Beads were resuspended by adding 500 µL of ammonium bicarbonate (AMBIC) solution at 50 mM to the columns after closing the outlets with caps and later transferred to low‐protein‐binding tubes (Eppendorf, #0030108116). This step was repeated, and the supernatant was pooled with the first in the same tube. Tubes containing the beads were centrifuged at 1000×*g* for 5 min, and all but 200 µL of the supernatant was carefully discarded. Afterwards, 0.5 µg of Trypsin Gold (Promega, #V5280) was added to each tube and incubated ON at 37°C in a thermoshaker. The following day, the supernatants were collected in fresh tubes after centrifugation at 1000×*g* for 5 min. The beads were rinsed with an additional 500 µL of LC‐MS grade water. After centrifugation, the supernatants from the first and second elution steps were combined, acidified with 2 µL trifluoroacetic acid (TFA), and desalted using Sep‐Pak tC18 solid‐phase extraction cartridges (Waters, #186002318). Water with 0.1% TFA was used as the equilibration and wash solvent, and 50% acetonitrile with 0.1% TFA was used as the elution solvent. Samples were lyophilized and diluted with 0.1% formic acid to reach a peptide concentration of 0.2 µg/µL before loading into glass vials for LC‐MS analysis.

### 4.12 Sample Preparation for Whole Proteome Analysis

A total of 2 × 10⁶ GM‐DCs were seeded into six‐well plates and stimulated with 1 µM CpGB for 4 h. All samples (WT: *n* = 4; *Zdhhc9* ^KO^: *n* = 3) were processed in technical replicates. After incubation, cells were harvested and washed three times with PBS. Subsequently, the pellets were transferred to low‐protein‐binding tubes, resuspended in 300 µL of MS‐grade lysis buffer (7 M urea, 2 M thiourea, 2% CHAPS, and 5 mM DTT), and sonicated for 15 min as described previously. Subsequently, lysates were centrifuged at 16,200×*g* at 4°C for 15 min. Then, protein levels were measured in the supernatant using the Pierce 660 nm protein assay. Protein lysates containing 20 µg of protein were transferred into Nanosep spin filter columns with 30 kDa MWCO Omega membranes and centrifuged at 16200×*g* for 15 min at RT. Flow‐throughs were discarded, and columns washed using equilibration buffer (8 M urea in 0.1 M Tris pH = 8.5). The first step of reduction with 8 mM DTT at 56°C for 15 min was performed. After that, the columns were washed twice with equilibration buffer and incubated with IAA for 20 min at RT, followed by two additional washing cycles. A second incubation step with DTT was conducted as described previously and continued by two washes with equilibration buffer and three additional washes with 50 mM AMBIC. Next, 0.4 µg of trypsin in 40 µL of 50 mM AMBIC was applied to the membranes of the columns and left ON at 37°C in a wet chamber. The next day, the concentration units were transferred into 1.5 mL tubes containing 10% trifluoroacetic acid and centrifuged as described. Another 40 µL of AMBIC buffer was added, and the flow‐through was collected after centrifugation. Samples were adjusted to 0.2 µg/µL as described above and stored at −80°C.

### 4.13 Analysis of Proteins via Liquid Chromatography–Mass Spectrometry (LC‐MS/MS) and Raw Data Processing

LC‐MS/MS analysis was conducted according to previous reports [85]. Tryptic peptides were separated using an Ultimate 3000 RSLCnano system (Thermo Fisher Scientific) with a PEPMAP100 C18 trap (5 µm, 0.3 × 5 mm) and an HSS‐T3 C18 analytical column (1.8 µm, 75 µm × 250 mm, Waters). Mobile phases consisted of 0.1% FA and 3% DMSO in water (A) or acetonitrile (B). Peptides were separated over 40 min (2%–35% B) at 300 nL/min and 55°C, with a total run time of 60 min. Eluting peptides were analyzed on an Orbitrap Exploris 480 (Thermo Fisher Scientific) in Top10 DDA mode. Full MS scans (m/z 350–1500) were acquired at 120,000 resolution at m/z 200 with a 300% AGC target and 50 ms maximum injection time. MS2 scans used a 1.6 m/z isolation window, 30% NCE, 15000 resolution, and a 25 ms maximum injection time. Spray voltage was 1.8 kV, funnel RF 40, and capillary temperature 275°C. Data were collected in profile mode with positive polarity. MS raw data were searched with MaxQuant (v2.0.3.0) against a UniProtKB/Swiss‐Prot [86] mouse protein reference database (release 2020_01, 17033 entries) and 172 common MS contaminants. Trypsin was set as a protease tolerating a maximum of two missed cleavages. The minimum peptide length was set to seven amino acids. Carbamidomethylation of cysteine was selected as fixed, whereas methionine oxidation and protein N‐terminal acetylation were set as variable modifications. Resulting peptide and protein identifications were filtered to a false discovery rate (FDR) of 1%. Match‐between‐runs was enabled using default parameters. For whole‐proteome analysis, label‐free quantification (LFQ) was performed using the standard MaxLFQ algorithm (LFQ minimum ratio count = 2, experiment‐wide cross‐run normalization enabled). Regarding the S‐palmitoylated proteome, LFQ was enabled with relaxed settings (LFQ minimum ratio count = 1, cross‐run normalization within parameter groups, that is, 17‐ODYA‐labeled samples and DMSO controls). The proteingroup.txt file generated was uploaded into Perseus (v2.3.9.0). Proteins that were potential contaminants and decoys were removed by site identification. Raw intensities were transformed to log2 values, and proteins with more than three values returning “NaN” after transformation were removed. Missing values were imputed from the normal distribution of the remaining values. Statistical analysis was performed using a two‐sample *t*‐test, FDR = 5 %, and s0 = 0.1. Data were exported to Excel for the generation of plots and figures. Enriched S‐palmitoylated proteins were identified using the SwissPalm tool. Both datasets were visualized in R (v4.4.1) using the RStudio interface (v2024.04.2). The comparison included 17‐ODYA versus DMSO in resting GM‐DCs (17‐ODYA/DMSO), 17‐ODYA with or without CpG‐B stimulation to assess TLR9‐driven remodeling (17‐ODYA CpGB/resting), and CpGB‐stimulated *Zdhhc9* ^KO^ versus WT cells to evaluate genotype‐dependent effects (CpGB KO/WT). Protein sets were subjected to pathway enrichment analysis using IPA (v24.0.2) or the gprofiler2 package (v0.2.3), with input based on their UniProt identifiers. Filtering thresholds for log_2_ fold changes (log_2_FC) and FDR are specified in the figures and legends. S‐palmitoylation site prediction was performed using GPS software, based on protein FASTA sequences retrieved from curated *Mus musculus* entries in the UniProtKB/Swiss‐Prot database. For simplicity, proteins were labeled using the names of their corresponding coding genes in all the figures.

### 4.14 RNA Isolation, cDNA Synthesis, and qPCR

Cell pellets ranging from 1–2 × 10^6^ cells were washed three times with PBS and lysed with 300 µL of TRIzol reagent (Invitrogen, #15596018). Subsequently, RNA was isolated using the Direct‐zol RNA Microprep Kit (Zymo Research Europe, #R2062) following the manufacturer's instructions, and RNA concentration and purity were assessed using a spectrophotometer (NanoDrop One, Thermo Scientific). Samples with an A260/280 ratio between 1.8 and 2.3, and without phenol contamination, were selected for downstream steps. Next, 200 ng of total RNA was used to generate cDNA. A mixture containing the RNA template, 1 µL oligo(dT) (Invitrogen, #18418020), and 1 µL dNTP mix (Invitrogen, #R0192) in RNase/DNase‐free water (Invitrogen, #10977049) was first subjected to a single pre‐annealing cycle (5 min 70°C à cold‐down to 4°C) using a thermocycler (ProFlex PCR System, Thermo Scientific). Immediately after, 5X First‐Strand Buffer (Invitrogen, 18080–093), DTT (Invitrogen, #18080‐093), RNase Inhibitor (Invitrogen, #10777‐019), and SuperScript III RT (Invitrogen, #18080‐093) were added to the reaction, gently mixed, and incubated at 55°C for 60 min in the thermocycler. Lastly, the reaction was inactive after heating at 70°C for 15 min. Dilution of the cDNA and primers was made in RNase/DNase‐free water. All the primers used in this study were purchased from IDTTM and are listed below: *Gapdh (F‐GGTTGTCTCCTGCGACTTCA, R‐CCCTAGGCCCCTCCTGTTAT); Zdhhc2 (F‐CTACTACGCCTACGCCATCC, R‐TCCAGCAATTCTTTCTCTGCAT); Zdhhc3 (F‐CTGGTTCCATCCCGAGACTAC, R‐CTCGATGAACTCTTTAGTGGCAT); Zdhhc6 (F‐CATAGCCCTGGGTGTTATAGCA, R‐CCTGAGACTTTTCCGGTTTCC); Zdhhc9 (F‐AAGGTGACACGGAAATGGGAG, R‐CGACACTCGAAGGCAAAGAA); Zdhhc20 (F‐GGAAAGACCGTTGTTTACCTTGT, R‐ACTCCTTCTCATAACGCTCCTTC); Myd88 (F‐ TGGCCTTGTTAGACCGTGA, R‐AAGTATTTCTGGCAGTCCTCCTC)*. Reaction cocktail with cDNA template, primers dilutions, and iQ SYBR GREEN Supermix (BioRad, #1708886) was prepared and loaded into optical reaction plates (Thermo Fisher Scientific, # 4346906). Quantification of gene expression was executed on a real‐time PCR thermal cycler (qTOWER iris, Analytik Jena) with the following cycling conditions: 95°C for 10 min (initial denaturation, 1 cycle), followed by 40 cycles of 95°C for 30 s, 58°C for 30 s, and 72°C for 1 min. In addition, the melting curve for each gene fragment was recorded. Outputs were analyzed and compared according to the formula 2^−ddCt^.

### 4.15 Analysis of Bulk‐RNAseq and Sc‐RNAseq Datasets

The datasets explored in this work were retrieved from GEO [87], including three bulk‐RNAseq studies (GSE188992, GSE106715, and GSE153392) and one sc‐RNAseq (GSE189120). Raw count matrices from bulk data were processed following the DESeq2 (v1.44.0) pipeline in RStudio. PAT‐coding genes were examined in the normalized matrix, and only those with a minimum of ten transcript counts per condition were visualized using the pheatmap package (v1.0.12). For the single‐cell data, the .h5 files were merged and converted into a format compatible with the Seurat (v5.3.0) workflow. Data cleaning criteria were applied in accordance with the original authors, down‐sampling cells that expressed more than 200 genes and exhibited less than 10% mitochondrial gene content. Afterward, the cleaned data were normalized, scaled, and dimensionally reduced following standard practices. In addition, data integration was implemented with Harmony (v1.2.3), and S‐palmitoylation Gene Scores were computed using the AddModuleScore function from the Seurat package. The input feature set consisted of the following genes: *Zdhhc1, Zdhhc2, Zdhhc3, Zdhhc4, Zdhhc5, Zdhhc6, Zdhhc7, Zdhhc8, Zdhhc9, Zdhhc11, Zdhhc12, Zdhhc13, Zdhhc14, Zdhhc15, Zdhhc16, Zdhhc17, Zdhhc18, Zdhhc19, Zdhhc20, Zdhhc21, Zdhhc22, Zdhhc23, Golga7*. Lastly, different plots were generated and compared across experimental conditions.

### 4.16 Cytokine Determination in Culture Supernatants

The culture supernatant was collected 4 h after TLR activation, otherwise specified. Samples were stored at ‐80°C until used. Cytokine concentration for IL‐12p70 (R&D, #DY419), IL‐10 (R&D, #DY417), IL‐6 (R&D, #DY406), TNFα (R&D, #DY410), and IFNβ (R&D, #DY406) was directly assessed by ELISA. In the case of IL‐1β, M‐CSF–differentiated BMMs were preincubated with or without 100 µM 2‐BP for 2 h, followed by stimulation with 100 ng/mL LPS for 4 h. To induce IL‐1β secretion, 1 mM neutralized ATP (Sigma‐Aldrich, #A6419) was added to the culture 45 min before the end of LPS stimulation. Culture supernatants were collected, and IL‐1β concentrations were determined by ELISA (R&D Systems, #DY401). Optical density (OD) was measured using a microplate reader (BioTek Cytation 5, Agilent), and the differential absorbance (ΔOD_450–570_) was recorded.

### 4.17 Statistical Analysis

The animal experiments included 3–6 animals per group, and data were presented as mean ± standard deviation (SD) in the corresponding result sections. The results showed representative data from at least three independent experiments, with the number of biological replicates provided in the corresponding figure legends. All statistical analyses were performed using GraphPad Prism software (version 10.3.1). The specific statistical tests used, unpaired *t*‐tests and one‐ or two‐way ANOVA, are detailed in the figure legends. When applicable, multiple testing correction was performed using the Benjamini–Hochberg method to control the false discovery rate (FDR). FDR‐adjusted *p*‐values (*q*‐values) below 0.05 were considered statistically significant and are reported in the figures.

## Author Contributions

**Juan N. Quiroz**: Data collection, analysis, and interpretation; manuscript writing, editing, and review. **Malte Sielaff**: Data collection, analysis, and interpretation. **Daria Kondrateva**: Data collection and analysis; manuscript review. **Fatima Boukhallouk**: Data collection. **Gloria J. Godoy**: Data collection. **Cecilia R. Molina**: Data analysis. **Brecht Moonen**: Data collection, analysis, and interpretation; manuscript editing and review. **Claudia C. Motran**: Manuscript editing and review. **Jeroen Bogie**: Manuscript editing and review. **Hugo D. Luján**: Data analysis; manuscript editing and review**. Stefan Tenzer**: Data analysis and interpretation; manuscript editing and review. **Tim Sparwasser**: Development and conception of the project, provision of resources, evaluation, and interpretation of the data; manuscript editing and review. **Luciana Berod**: Development and conception of the project, provision of resources, and evaluation and interpretation of the data; mentoring and supervision; manuscript writing, editing, and review.

## Conflicts of Interest

The authors declare no conflicts of interest.

## Peer Review

The peer review history for this article is available at https://publons.com/publon/10.1002/eji.70039.

## Supporting information




**Supporting File 1**: eji70039‐sup‐0001‐SuppMat.pdf

**Supporting File 2**: eji70039‐sup‐0003‐TableS2.xlsx

**Supporting File 3**: eji70039‐sup‐0004‐TableS3.xlsx

**Supporting File 4**: eji70039‐sup‐0005‐TableS4.xlsx

**Supporting File 5**: eji70039‐sup‐0005‐TableS4.xlsx

**Supporting File 5**: eji70039‐sup‐0006‐TableS5.xlsx

## Data Availability

The MS proteomics data have been deposited to the ProteomeXchange Consortium (http://proteomecentral.proteomexchange.org) via the PRIDE [88] partner repository with the dataset identifier PXD066223.
