# Supplementary material for: TLR9‐Driven S‐Palmitoylation in Dendritic Cells Reveals Immune and Metabolic Protein Targets
Source: Eur J Immunol. 2025 Aug 19;55(8):e70039. doi: 10.1002/eji.70039 (PMC12363430; doi:10.1002/eji.70039)
Supplement: Supplementary file 1 — Supporting File 1: eji70039‐sup‐0001‐SuppMat.pdf [file EJI-55-e70039-s001.pdf]

Supplementary Figure S1

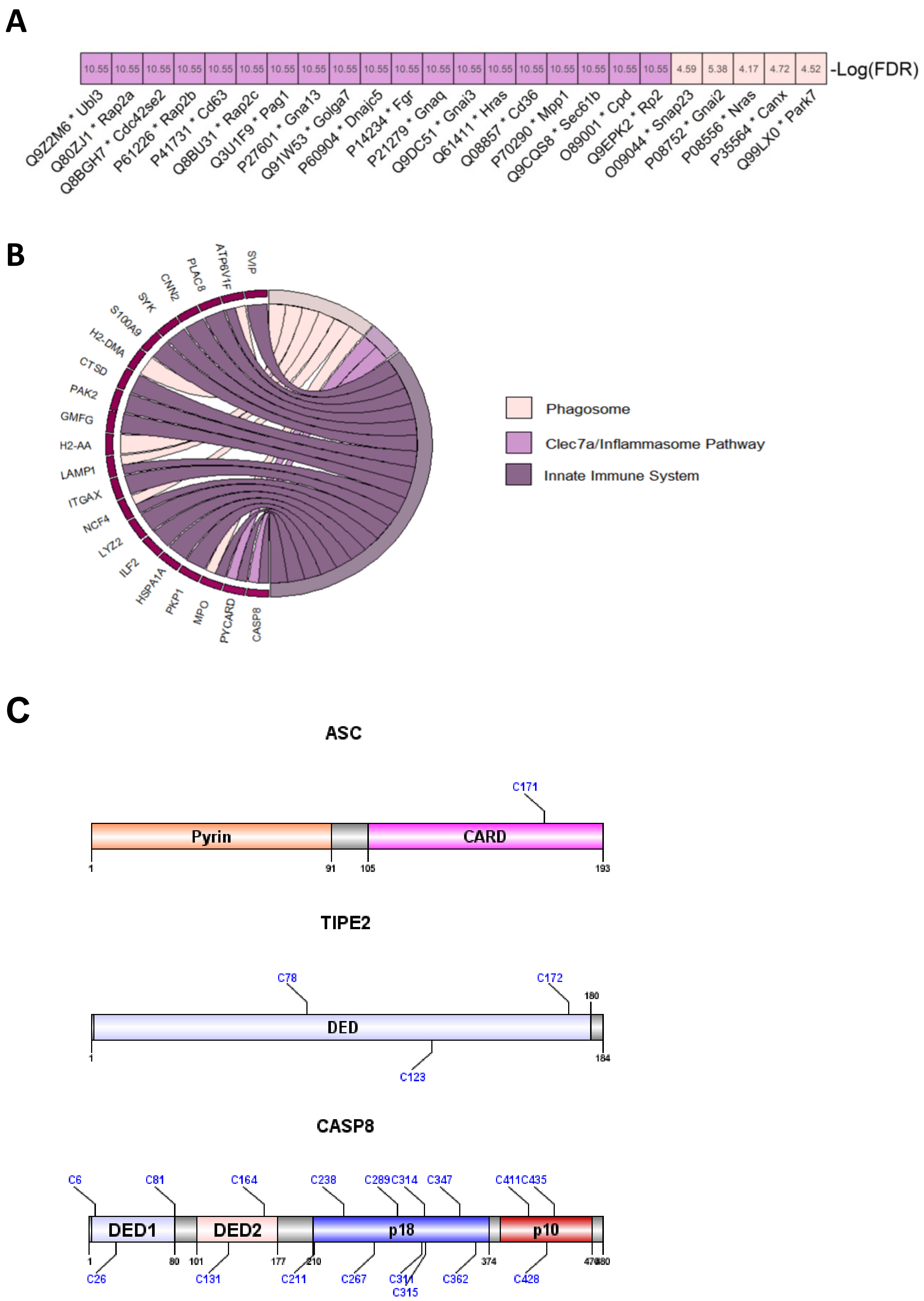

Supplementary Figure S1: Palmitoyl-proteome of homeostatic GM-DCs. Resting bulk GM-CSF cultures were generated from the bone marrow of C57BL/6 mice. Cells were then incubated with 17-ODYA or vehicle (DMSO) for 4 hours, followed by protein extraction and processing for click chemistry-based enrichment of S-palmitoylated proteins and MS identification (17-ODYA/DMSO). **(A)** S-palmitoylated proteins in our dataset annotated as palmitate-modified in UniProtKB. Proteins are presented with both UniProt identifiers and gene names, order by log_2FC (17-ODYA/DMSO) **(B)** Chord diagram with novel S-palmitoylated proteins identified in this study and their association with immune-related functions. **(C)** Predictions of S-palmitoylated sites in ASC, TIPE2 and CASP8 using the GPS algorithm. Protein domains were annotated according to Swiss-Prot, and cysteines positions highlighted.

Supplementary Figure S2

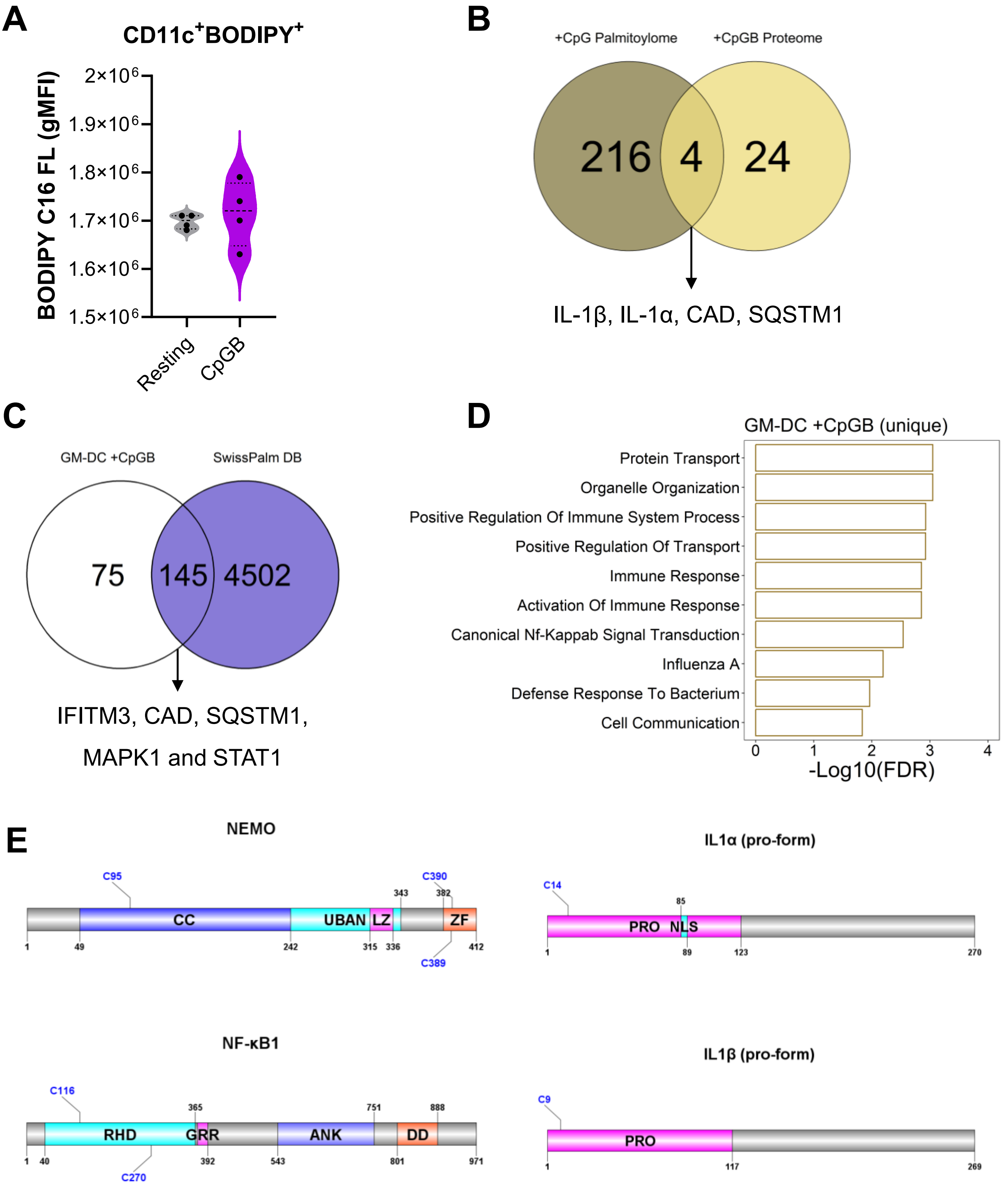

Supplementary Figure S2: CpGB-induced GM-DC maturation impact on S-palmitoylation dynamics. Bulk GM-CSF-derived cultures were stimulated with 1 µM CpGB for 4 hours or left unstimulated (resting) in the presence of 17-ODYA, and subject to S-palmitoylated proteome analysis using click-MS. In parallel, resting and CpGB–activated cultures were processed for whole proteome profiling via MS. **(A)** FACS measurements of BODIPY C16 FL incorporation within CD11c$^+$ cells at resting and after 4 hours of activation with 1 µM CpGB. **(B)** Proteins with increased protein levels and S-palmitoylated rates during CpGB stimulation. **(C)** Venn diagram showing the overlap between identified lipid-modified targets and previously reported entries in the SwissPalm database. **(D)** Biological processes found to be enriched in the dataset of 75 newly identified S-palmitoylated proteins during TLR9 stimulation. **(E)** GPS-based predictions of S-palmitoylated sites in novel protein targets regulated during TLR9 activation. Protein domains were annotated based on Swiss-Prot, and putative cysteines highlighted. For the S-palmitoylated proteome analysis, three independent biological replicates (n = 3) were evaluated in a single run, each analyzed in technical triplicates. Regarding whole proteome, all samples (n = 4) were processed in technical duplicates. The dataset generated after raw data processing (see Methods) was subjected to downstream analysis based on proteins meeting the following criteria: $|\log_2 FC| > 1$ and FDR < 0.01. Experiments involving DC maturation included 3–4 biological replicates, assessed in three independent runs. Data are presented as mean ± SD. Unpaired parametric t-tests were used to compare resting and CpGB–stimulated DCs (p-value <0.05).

Supplementary Figure S3

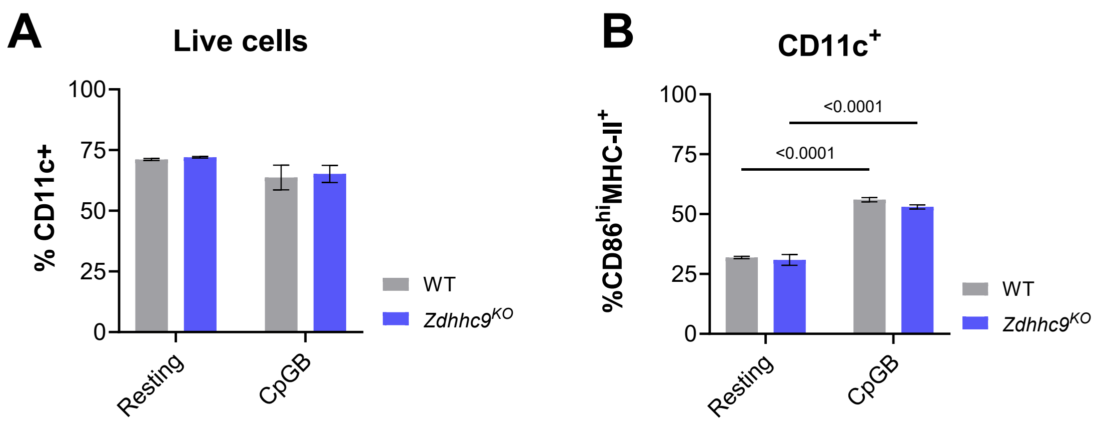

Supplementary Figure S3: GM-DC viability, differentiation, and maturation are preserved in *Zdhhc9*-deficient mice. GM-DC cultures generated from *Zdhhc9*^{KO} or WT mice were stimulated with 1 μM CpGB for 4 hours or left unstimulated (resting). The percentage of viable CD11c^{+} cells and CpGB–induced maturation was assessed by FACS and contrast across genotypes. **(A and B)** FACS assessment of DC differentiation (% of CD11c+) and activation (% of CD86^{hi}MHC-II^{+}) of resting and 4-hour CpGB stimulated GM-DC cultures. Ordinary two-way ANOVA followed by multiple comparisons with FDR correction (at a 0.05 significance level) was used to compare genotypic- and activation-related differences. Results include biological replicates (WT: n = 3; *Zdhhc9*^{KO}: n = 3) and are representative of three independent experiments. Statistical analysis was performed using ordinary two-way ANOVA followed by multiple comparisons with FDR correction at a 0.05 significance level. Only statistically significant changes are shown (q-value<0.05).

Supplementary Figure S4

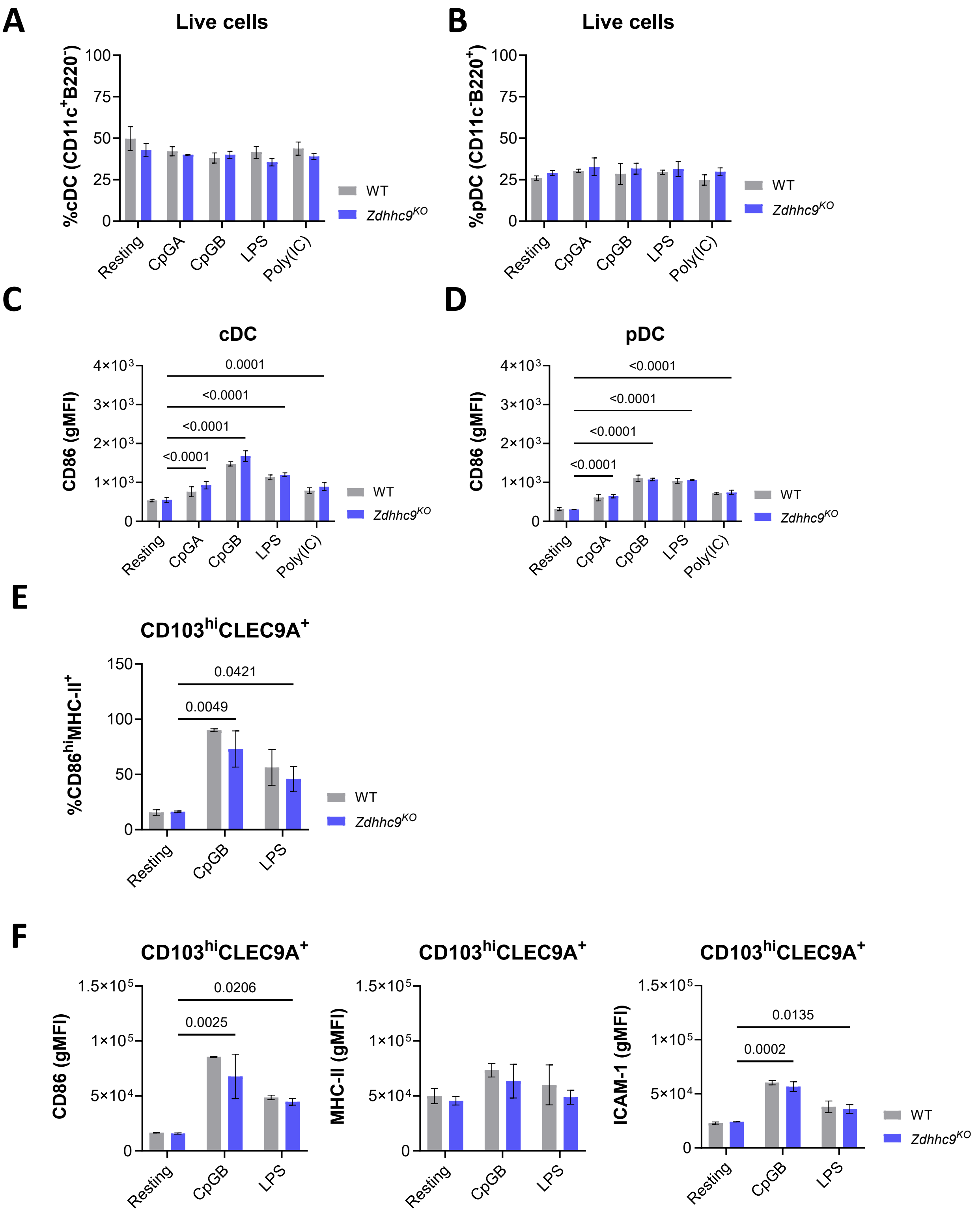

Supplementary Figure S4: *In vitro* differentiation, and maturation of Flt3L- and CD103+ DCs were comparable between WT and *Zdhhc9*^{KO} mice. DC cultures generated from *Zdhhc9*^{KO} or WT mice were stimulated with different TLR agonists for 4 hours (1 µM CpGB; 1 µM CpGA; 100 ng/mL LPS; 50 µg/ml PolyIC), or left unstimulated (resting). Markers of DC maturation were evaluated and compared between genotypes. **(A and B)** Genotype-dependent differences in the % of live cDC and pDC, and **(C and D)** CD86 expression evaluated in Flt3L-DC cultures that were stimulated or not with distinct TLR agonists for 4 hours (1 µM CpGA; 1 µM CpGB; 100 ng/ml LPS and 0.1 µg/ml Poly(I:C)). **(E)** % of CD103^{hi}CLEC9A^{+} expressing CD86 and MHC-II inspected in CD103^{+} DC cultures at resting and upon 6 hours stimulation with 100 ng/ml LPS or 1 µM CpGB. **(F)** Average expression of activation markers on CD103^{hi}CLEC9A^{+} populations. Ordinary two-way ANOVA followed by multiple comparisons with FDR correction (at a 0.05 significance level) was used to compare experimental groups. Results include biological replicates (WT: n = 3; *Zdhhc9*^{KO}: n = 3) and are representative of three independent experiments. Statistical analysis was performed using two-way ANOVA followed by multiple comparisons with Benjamini–Hochberg. For clarity, only significant comparisons between *Zdhhc9*^{KO} experimental conditions (q-value <0.05) are reported.

Supplementary Figure S5

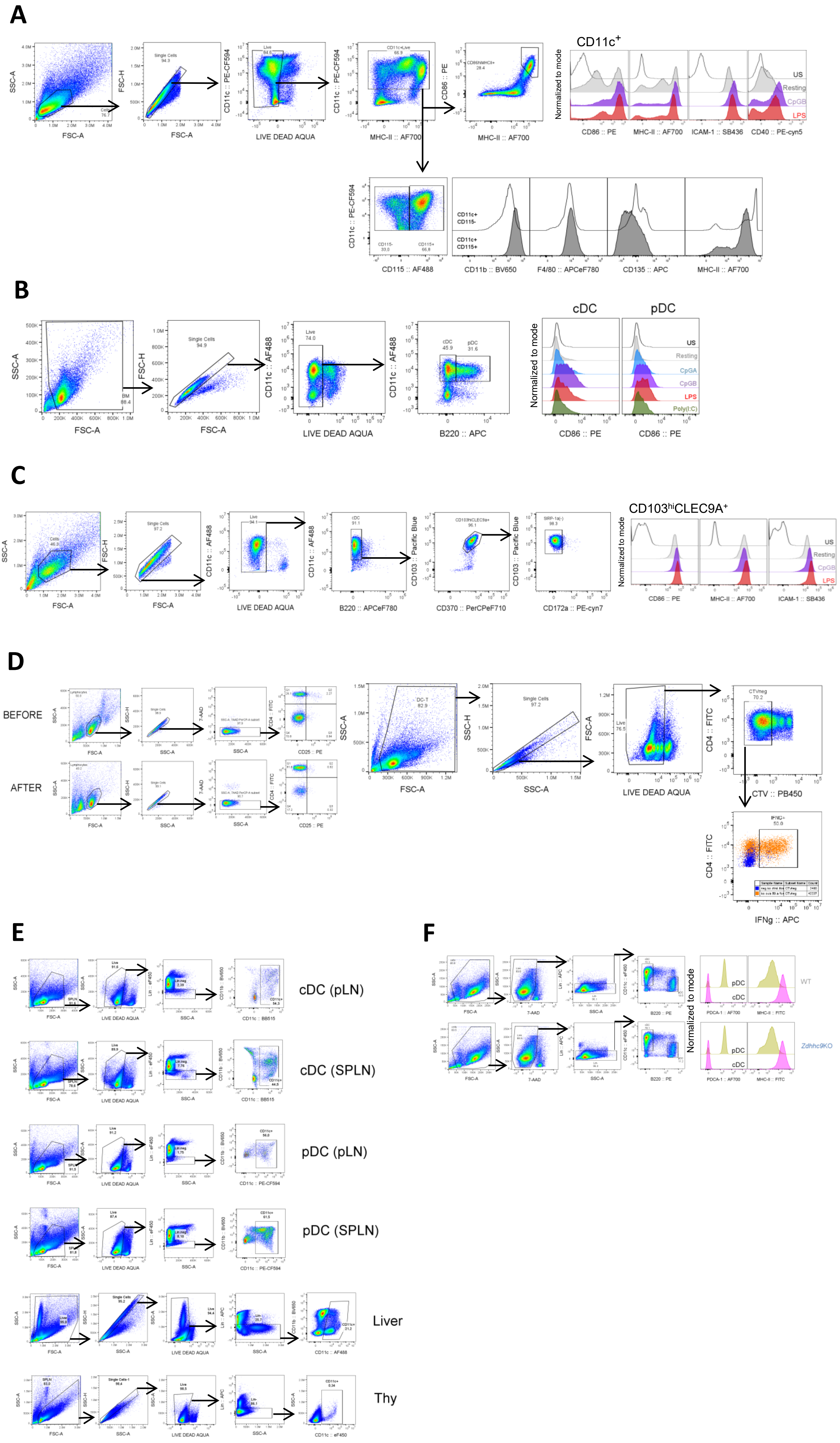

Supplementary Figure S5

Supplementary Figure S5: Phenotypic characterization of DC cultures, tissue-derived DCs and CD4+ T cell activation by FACS. Gating strategy for the identification of **(A)** total CD11c^{+} cells, mature DCs (CD86^{hi}MHC-II^{+}), and a DC-like subset (CD11c^{+} CD115^{-} CD135^{hi} F4/80^{low}) within GM-CSF-derived bone marrow cultures; **(B)** cDC and pDC subsets within Flt3L-DC cultures; and **(C)** *in vitro* generated CD103^{hi}CLEC9A^{+} DCs. **(D)** Enrichment of total CD4^{+} T cells from pooled SPLN/pLNs of mice. Plots of pre- and post-enrichment of CD4^{+} are shown. The evaluation of CD4^{+} T cell proliferation, IFNγ production and activation markers was assessed following 4-days co-culture with DCs. **(E)** Phenotypic characterization of tissue-derived DCs from SPLN, pLNs, liver and Thy. Distinct antibody panels were designed to inspect cDCs, pDCs or total CD11c-expressing cells. Doublet and dead cell exclusion were implemented in all the analysis, and lineage negative (Lin^{-}) selection applied when analyzed tissue-derived DCs. Lineage markers for cDCs and pDCs include CD3, CD19, NK1.1, and F4/80. Additionally, CD64 was incorporated for liver- and Thy-derived DCs. **(F)** Evaluation of cDC and pDC subsets derived from splenocyte cultures. After selection of lineage negative populations, CD11c and B220 expressions were evaluated. SPLN-derived cDCs were gate within CD11c$^{+/hi}$B220^{-} population whereas pDCs express CD11c$^{+/int}$B220^{+}. The gating strategy was further validated by evaluating PDCA-1 and MHC-II expression, indicative of pDCs and cDCs, respectively.

Supplementary Tables S1-5

Supplementary Table S1: S-palmitoylated proteome in resting GM-DCs. A total of 550 S-palmitoylated proteins were identified in resting GM-DC cultures. Changes in protein abundance in 17-ODYA-treated cultures was assessed relative to vehicle (DMSO) controls and quantified as log_2FC based on LFQ values. The dataset includes UniProt identifiers, protein names, corresponding gene symbols, and statistical metrics (FDR and log_2FC) for each protein. Only proteins with FDR < 0.01 were included in the analysis.

Supplementary Table S2: Subset of S-palmitoylated proteins identify with high and medium confidence in resting DC cultures. Proteins were classified as high confidence when their relative abundance in 17-ODYA–treated samples was at least 10-fold higher compared to DMSO controls. Medium confidence proteins were defined by a minimum 5-fold increase in abundance relative to DMSO.

Supplementary Table S3: STRING predicted interactions of novel S-palmitoylated targets in resting GM-DCs. Novel lipid-modified proteins were uploaded to STRING-db for protein-protein interaction analysis. Only high-confidence interactions were included, based on the STRING combined interaction score. Both combined and individual scores are provided for each protein-protein interaction.

Supplementary Table S4: Analysis of differentially abundant S-palmitoylated proteins in GM-DCs during CpG-B stimulation. S-palmitoylated proteome of CpGB-activated DCs compared to unstimulated (resting) conditions. Changes in protein abundance were considered statistically significant when FDR < 0.01.

Supplementary Table S5: Proteins with altered S-palmitoylation in Zdhhc9-deficient GM-DCs during TLR9 activation. The list of overrepresented S-palmitoylated proteins in CpGB–activated WT GM-DCs was compared to the S-palmitoylated proteome of stimulated GM-DCs from $Zdhhc9^{KO}$ mice. Targets with impaired S-palmitoylation due to *Zdhhc9* deficiency were identified based on the following criteria: (1) proteins enriched in WT GM-DCs upon CpGB stimulation but unchanged in $Zdhhc9^{KO}$ GM-DCs, or (2) proteins enriched in WT GM-DCs during CpGB stimulation that exhibited reduced S-palmitoylation in *Zdhhc9*-deficient DCs.

Supplementary Table S6

Antibodies				
Antigen	*Fluorochrome*	*Clone*	*Company*	*Catalog*
CD11c	Alexa Fluor 488	N418	Invitrogen	53-0114
CD11c	PE-CF594	N418	BD Horizon	565591
CD11c	eF450	N418	Invitrogen	48-0114
CD11c	BB515	B-ly6	BD Horizon	564490
CD11b	BV650	M1/70	Biolegend	101259
MHC-II (I-A/I-E)	Alexa Flour 700	M5/114.15.2	Biolegend	107622
MHC-II (I-A/I-E)	eFluor450	AF6-120.1	Invitrogen	48-5320
MHC-II (I-A/I-E)	FITC	M5/114.15.2	Invitrogen	11-5321
CD86	PE	GL1	Invitrogen	12-0862
CD103	Pacific Blue	2E7	Biolegend	121418
CD103	APC	2E7	Invitrogen	17-1031
CD103	PE	2E7	Invitrogen	12-1031
CD115	Alexa Fluor 488	AFS98	Biolegend	135512
CD135	APC	A2F10	eBioscience	17-1351
CD370 (CLEC9A)	PerCPeFluor710	42D2	Invitrogen	46-5975
CD172a (SIRP-1a)	PE/Cyanine7	P84	Biolegend	144008
CD45R (B220)	APC	RA3-6B2	Cytek Tonbo	20-0452
CD45R (B220)	APCeFluor780	RA3-6B2	Invitrogen	47-0452
CD45R (B220)	PE	RA3-6B2	Invitrogen	12-0452
CD54 (ICAM-1)	Super Bright 436	eBioKAT-1	Invitrogen	62-0542
CD40	PE/Cyanine5	1C10	Invitrogen	15-0401
CD4	FITC	RM4-5	Invitrogen	11-0042
CD25	PE	PC61.5	Invitrogen	12-0251
IFNγ	APC	XMG1.2	BD Horizon	554413
CD69	PE	H1.2F3	Invitrogen	12-0691
F4/80	eFluor450	BM8	Invitrogen	48-4801
CD3	eFluor450	17A2	Invitrogen	48-0032
CD19	eFluor450	eBio1D3	Invitrogen	48-0193
NK1.1	eFluor450	PK136	Invitrogen	48-5941
F4/80	APC	BM8	Invitrogen	17-4801
F4/80	APCeFluor780	BM8	eBioscience	47-4801
CD3e	APC	145-2C11	Invitrogen	17-0031
CD19	APC	eBio1D3	Invitrogen	17-0193
NK1.1	APC	PK136	Invitrogen	17-5941
CD64	APC	X54-5/7.1	Biolegend	139306
XCR1	PE	ZET	Biolegend	148204

Supplementary Table S6

CD8a	PerCP	53-6.7	Biolegend	100732
Siglec H	PerCPeFluor710	eBio440c	Invitrogen	46-0333
CD9	FITC	MZ3	Biolegend	124808
Ly-6A/E (Sca-1)	PE/Cyanine7	D7	Invitrogen	25-5981
Ly-6D	PE	49-H4	Biolegend	138604
CD317 (PDCA-1)	Alexa Flour 700	927	Biolegend	127038
CD199 (CCR9)	APC	eBioCW-1.2	Invitrogen	17-1991
**Probes and fluorescent dyes**				
Name	*Detector*	*Company*		*Catalog*
LIVE/DEAD™ Fixable Aqua Dead Cell Stain Kit	526 nm	Invitrogen		L34965
CellTrace™ Violet Cell Proliferation Kit	456 nm	Invitrogen		C34557
**Other reagents**				
Name		*Company*		*Catalog*
Dulbecco's Phosphate Buffered Saline Solution (PBS)		Sigma-Aldrich		D8537
Paraformaldehyde		Sigma-Aldrich		P6148
PBA-E<br>0.25%BSA, 0.02% sodium azide, 2mM EDTA in PBS		In-house made		
PBA-S<br>0.5% Saponin, 0.25%BSA, 0.02% sodium azide, 2mM EDTA in PBS		In-house made		
FC block (anti-CD16/CD32) from producing cell line (clone 2.4G2)		In-house made		

Supplementary Table S6: Antibodies and reagents used for FACS. List providing antigen, clone and suppliers' information for antibodies and reagents used in this study.
